# Genome-wide association study (GWAS) analyses of early anatomical changes in rose adventitious root formation

**DOI:** 10.1038/s41598-024-75502-1

**Published:** 2024-10-23

**Authors:** David Wamhoff, Annina Marxen, Bhawana Acharya, Monika Grzelak, Thomas Debener, Traud Winkelmann

**Affiliations:** 1 Institute of Horticultural Production Systems, Section Woody Plant and Propagation Physiology, Leibniz Universität Hannover, Hannover, Germany; 2 Institute of Horticultural Sciences, Section of Ornamental Plants, Doctoral School, Warsaw University of Life Sciences, Warsaw, Poland; 3 Institute of Plant Genetics, Section Molecular Plant Breeding, Leibniz Universität Hannover, Hannover, Germany

**Keywords:** Association mapping, Genome-wide association study, Histology, Phloem, Rooting, *Rosa* × *hybrida*, Xylem, Genome-wide association studies, Agricultural genetics, Natural variation in plants, Plant breeding, Plant morphogenesis, Plant regeneration, Plant physiology

## Abstract

Adventitious root (AR) formation is a genetically complex trait with high genotypic variability. Therefore, only a limited range of cultivars are currently propagated by cuttings in rose. In this study, we analysed the anatomy of in vitro shoots, the early formation of root primordia (RP) and the formation of ARs in a diverse set of 106 rose genotypes. Correlation analysis indicated that the growth in shoot diameter and the vasculature dimensions after 1 week of rooting contributed to successful AR formation. Using phenotypic data for genome-wide association studies (GWAS) analyses, nine significantly associated single nucleotide polymorphisms (SNPs) and genomic regions contributing to various RP and AR formation traits were identified. The contribution of genomic regions to trait variation was notably greater for traits associated with earlier processes than for traits associated with later developmental stages. The combination of RP and AR data allowed the detection of regions by GWAS that contain factors that potentially limit RP emergence. Homologues of 47 genes known to be involved in AR formation from the literature could be assigned to the identified peaks. Further studies are needed to investigate the suitability of SNPs exhibiting strong effects as allele-specific PCR markers for use in breeding.

**Supplementary Information:**

The online version contains supplementary material available at 10.1038/s41598-024-75502-1.

## Introduction

Roses have enormous economic importance in various horticultural market segments, such as garden plants, potted plants and especially cut flowers^1^. To meet various product and production demands, more than 30,000 cultivars within the genus *Rosa* L. show high diversity^2,3^. Modern rose cultivars are mainly tetraploid hybrids and are highly heterozygous^4^. To achieve true-to-type propagation, vegetative propagation methods are primarily applied^2,5,6^. Pot roses are propagated mainly by cuttings, and the ability to form adventitious roots (ARs) is an important goal in breeding cultivars for this segment^5^. However, the propagation of cut roses and garden roses still depends mainly on laborious and costly xenovegetative propagation methods, such as stenting and grafting^5,7^. In these groups, the ability for AR formation differs strongly between genotypes, and some genotypes do not form ARs at all^8–10^.

Successful root formation in vegetative propagation systems involves numerous processes, leading to the categorisation of AR formation into three essential phases: facultative dedifferentiation is followed in chronological order by the induction, initiation and expression phases^11,12^. The induction phase is characterised by changes at the cellular level, such as wound response and plant hormone signalling, while in the initiation phase, microscopically recognisable cell division activities result in the formation of root primordia (RP)^13–15^. Finally, the elongation of RP and root emergence through the shoot cortex occur at the expression phase^16^. Apart from many exogenous factors, the genetic ability to form ARs has been identified as the most significant prerequisite for successful rooting and can vary significantly even within a species^12,17^. Hundreds of genes specifically those involved in auxin transport and signalling, carbohydrate-associated genes or cell division-associated genes have been postulated to be involved in different phases of AR formation^17–19^. Rooting success is generally defined by considering the emergence of ARs and/or their number and length. However, this does not allow conclusions to be drawn about differences in the earlier processes of AR formation. For example, it has been reported that a high degree of lignification can hinder the initiated RP from emergence^20,21^.

Genome-wide association studies (GWASs) are powerful tools for analysing highly quantitative traits such as AR formation. For roses, the WagRhSNP 68 K Axiom SNP array comprising 68,893 single nucleotide polymorphisms (SNPs) is available^22^; this allows the identification of genomic regions involved in complex processes, such as AR formation, through high-density marker analysis. Previous GWASs have investigated AR formation in rose^8,10^. However, these studies considered macromorphological traits such as root number, root mass or the emergence of ARs but not histological characteristics or early events in RP formation. In addition, gene expression studies, e.g., in *Petunia* or *Populus*, have investigated differentially expressed genes involved in different phases of AR formation but have not compared multiple genotypes with genetic differences in AR formation^18,19^.

In this study, we present the results of comprehensive phenotypic and anatomical investigations of cuttings of 106 different rose genotypes rooted under in vitro conditions addressing the early phase of RP formation. RP and AR formation data were used to perform GWAS analyses to decipher the role of pivotal genomic regions on related traits of interest. Moreover, through the correlation of data regarding the anatomical architecture of shoot bases with RP and AR formation data, we addressed the question of associations between the inner shoot structure and the capability for AR formation.

## Results

### Root primordia formation

**Fig. 1:**
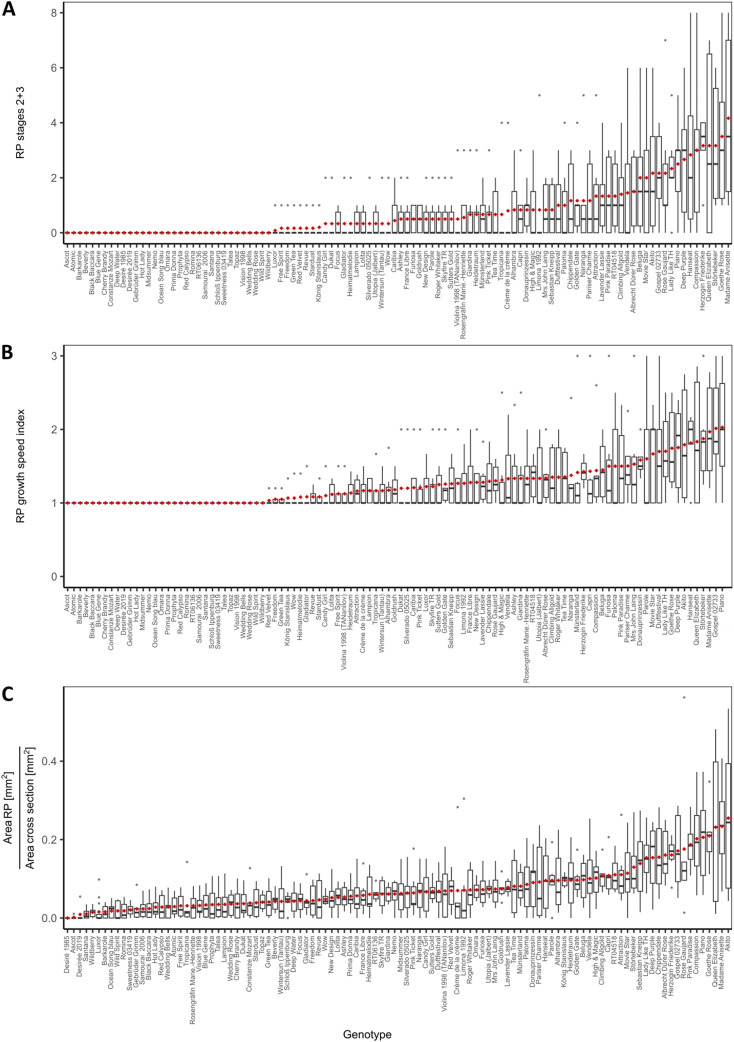
Root primordium (RP) formation characteristics of 106 tested rose genotypes after 1 week of incubation on hormone-free rooting media. (**A**) Number of RP at developmental stages 2 and 3, (**B**) RP growth speed index and (**C**) ratio of the total RP area to the cross-sectional area. Genotypes are ordered based on their means (◆). The replicate numbers per genotype are given in Supplementary Table S2.

To characterise RP formation in 106 rose genotypes, in vitro-generated shoots were cultivated for one week on hormone-free rooting medium and then harvested for histological analysis. RP formation was detected in all 106 genotypes, except for the genotype ‘Desiré 2006’, which did not show RP formation in any of the shoot bases analysed. The highest average number of RP per shoot was found for the ‘Compassion’ genotype, with 8.8 RP (Supplementary Fig. S1). To also consider the speed of RP formation, the RP were categorised into three developmental stages. All RP-forming genotypes had RP in early stage 1. Only 69 genotypes had RP in stage 2, and 44 had RP in the most developed stage 3 (Supplementary Fig. S1). As further developed RP stages 2 and 3 were of particular interest, their numbers were summed (Fig. 1A). Seventy-three genotypes formed RP of either stage 2 or 3 (Fig. 1A), with ‘Madame Anisette’ showing the greatest number of RP with 4.2 per shoot base (Fig. 1A). We calculated an RP growth speed index (Fig. 1B), which has a value of 1 when all RP were assigned to stage 1 and higher values when more RP of further developed stages were present. Accordingly, 73 genotypes showed an index value greater than 1, ranging between 1.03 (‘Red Velvet’) and 2.03 (‘Piano’) (Fig. 1B). Additionally, the area of RP per cross-section was measured, and its ratio to the total cross-sectional area was calculated (Fig. 1C). RP accounted for a maximum of 25% of the total cross-sectional area (‘Akito’; Fig. 1C).

### AR formation traits

The in vitro rooting experiments were conducted over a period of 3 weeks. Rooting was assessed weekly, and the number of roots per rooted shoot was determined using a destructive final evaluation after 3 weeks. The reference genotype ‘Vendela’ was included in as many rooting experiments as possible and showed interexperimental variation only for the rooting percentage after 2 weeks and the number of roots but not for AR formation after 1 and 3 weeks (Supplementary Fig. S2). After 1 week, 57 genotypes exhibited rooting, with percentages ranging from 1.1% (13 genotypes) to 53.3% (‘Sebastian Kneipp’) (Supplementary Fig. S3). After 2 weeks, strong progress was observed, with all the genotypes showing ARs and ‘Madame Anisette’ already reaching 100% rooting percentage (Supplementary Fig. S3). After 3 weeks, the rooting percentages ranged from 6.7% (‘Prophyta’) to 100% (‘Madame Anisette’; ‘Golden Gate’) (Supplementary Fig. S3). The average number of ARs per rooted shoot ranged from 1.4 (‘Prophyta’) to 7.9 (‘Madame Anisette’) (Supplementary Fig. S3).

**Fig. 2:**
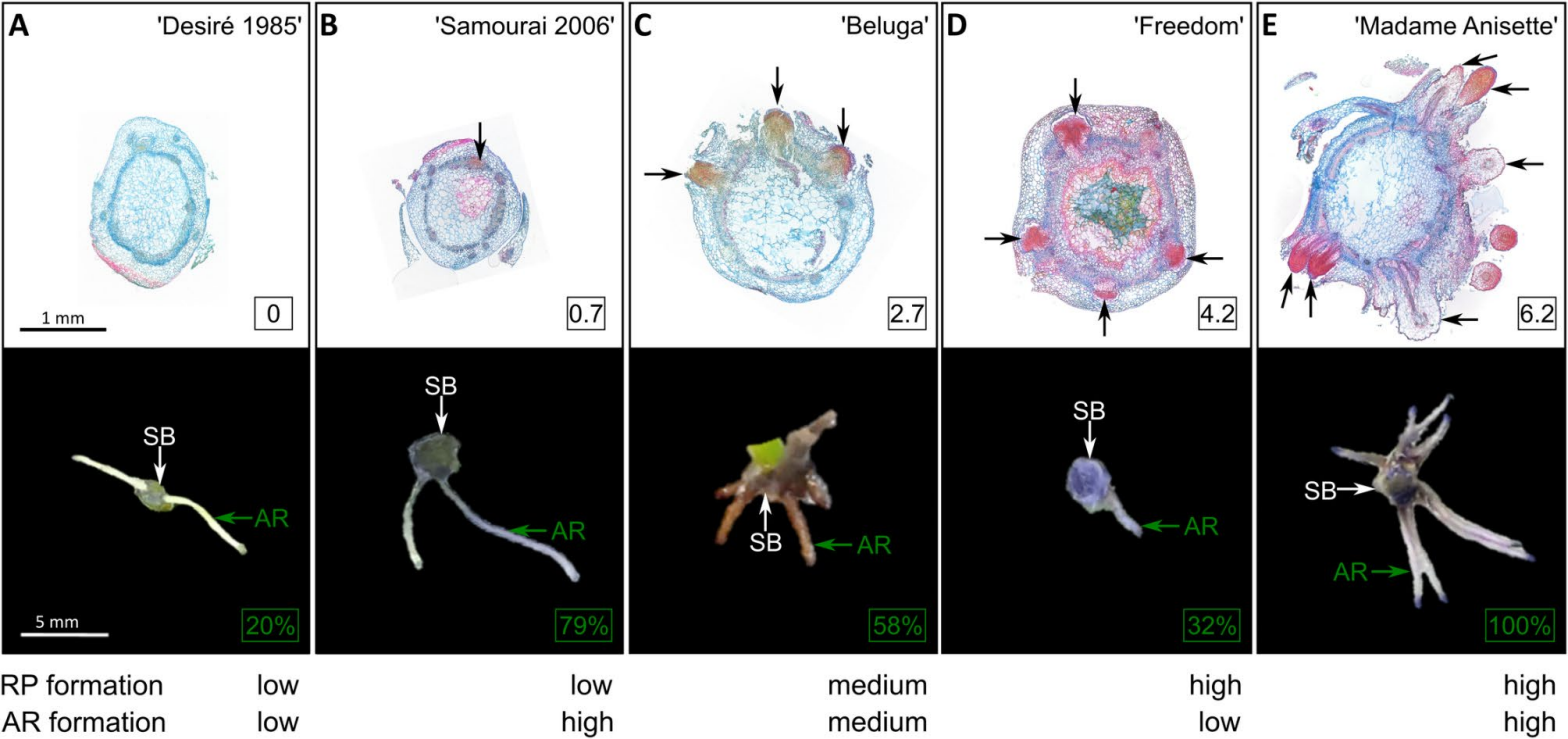
Characteristic phenotypes for root primordium (RP) and adventitious root (AR) formation as represented by selected genotypes. RP formation at different developmental stages evaluated on RAA-stained cross-sections after 1 week of rooting (upper row) with each RP structure in a cross-section indicated by a black arrow. The characteristic AR formation after 3 weeks of rooting is shown in the lower row; AR are marked with green arrows for a single representative example per genotype, and the shoot bases are marked with white arrows. Representative examples of genotypes with (**A**) low RP and AR formation, (**B**) low RP and high AR formation, (**C**) medium RP and AR formation, (**D**) high RP and low AR formation or (**E**) high RP and AR formation are presented. The values in squares show the mean number of RP per cross-section (upper row) and the mean rooting percentages after three weeks (lower row) per genotype. Abbreviations: AR, adventitious root; RP, root primordium; SB, shoot base.

Figure 2 exemplifies the characteristic RP and AR formation phenotypes, each represented by a typical representative genotype. One group of genotypes had low numbers of RP at 1 week and poor AR formation (‘Desiré 1985’); however, in other cases, low RP numbers and relatively good rooting responses (‘Samourai 2006’) at 3 weeks were observed (Fig. 2A, B). The genotype ‘Beluga’ is an example of both a medium number of RP and ARs (Fig. 2C). Interestingly, the ‘Freedom’ genotype developed many RP but showed only low numbers of ARs after 3 weeks (Fig. 2D), whereas the ‘Madame Anisette’ genotype exhibited both a high RP number and high AR formation (Fig. 2E).

### Anatomical shoot base characteristics of 106 rose genotypes

**Fig. 3:**
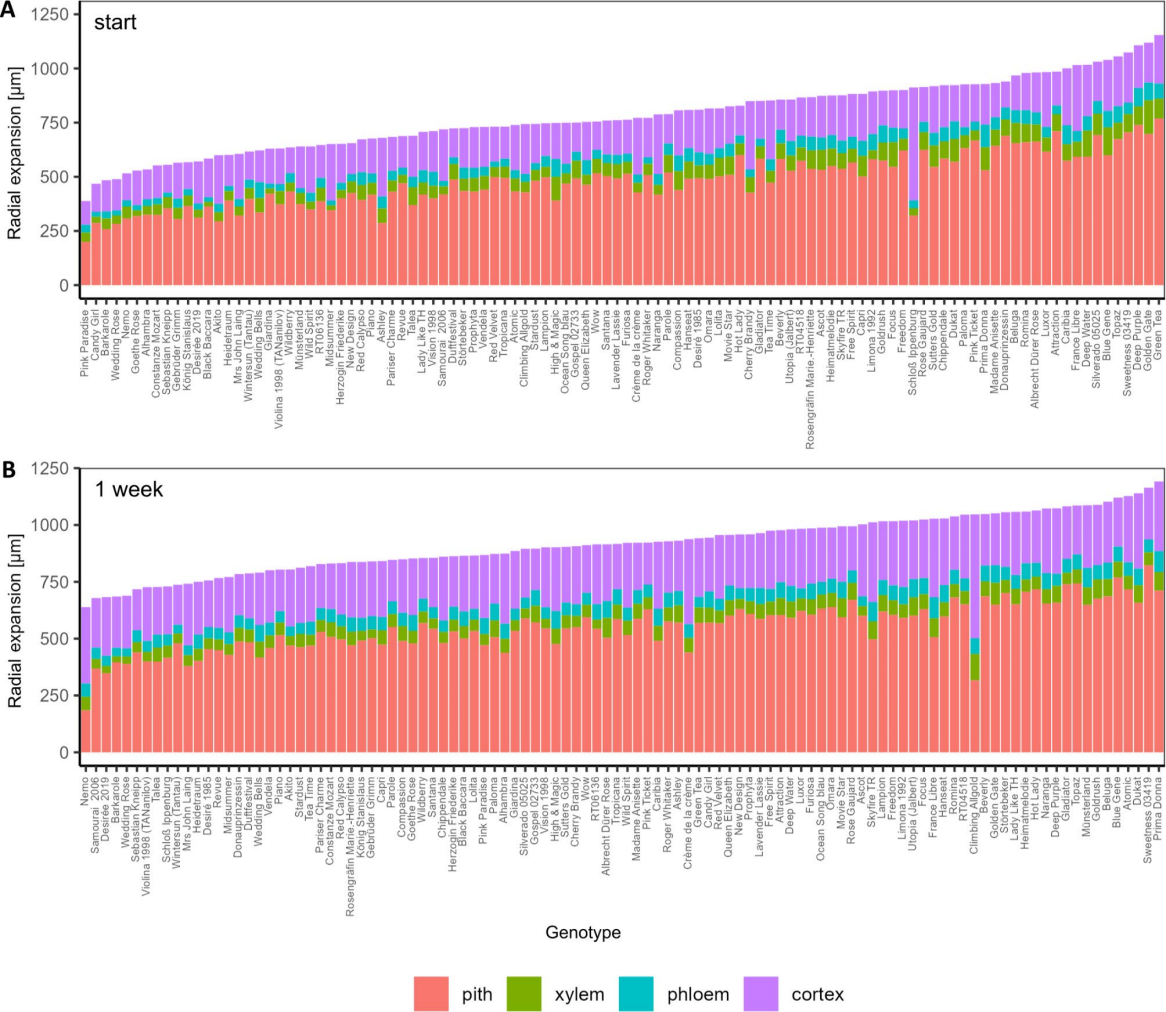
Radial expansion of tissues in shoot bases of 106 rose genotypes. The mean values for radial expansion of the pith, xylem, phloem and cortex are shown, which in sum result in the shoot radius. The values are presented for shoots at the beginning of the rooting experiment (start) and after 1 week of cultivation on hormone-free rooting medium (1 week). The mean values and standard deviations are shown in Supplementary Tables S2 and S3 for 1 week (*n* ≥ 5) and the start (*n* ≥ 2), respectively.

Radial expansions of the pith, xylem, phloem, and cortex were measured in cross-sections (CS) of the 106 tested rose genotypes at the beginning of rooting experiments and after 1 week of cultivation on rooting medium (Fig. 3). Genotypic variation was detected for all four tissue types. For the majority of genotypes, the pith contributed most to the shoot radius, both at the beginning of the experiments and after 1 week of cultivation (Fig. 3A, B). Only ‘Schloß Ippenburg’ (start), ‘Nemo’ (1 week) and ‘Beverly’ (1 week) stood out for their large expansion of the cortex (Fig. 3). The vascular tissues differed in their expansion among genotypes, and the phloem-xylem ratio ranged between 0.6 (‘Climbing Allgold’) and 1.7 (‘RT06136’) (Supplementary Table S2).

### Correlations of RP, AR and anatomical shoot parameters

**Fig. 4:**
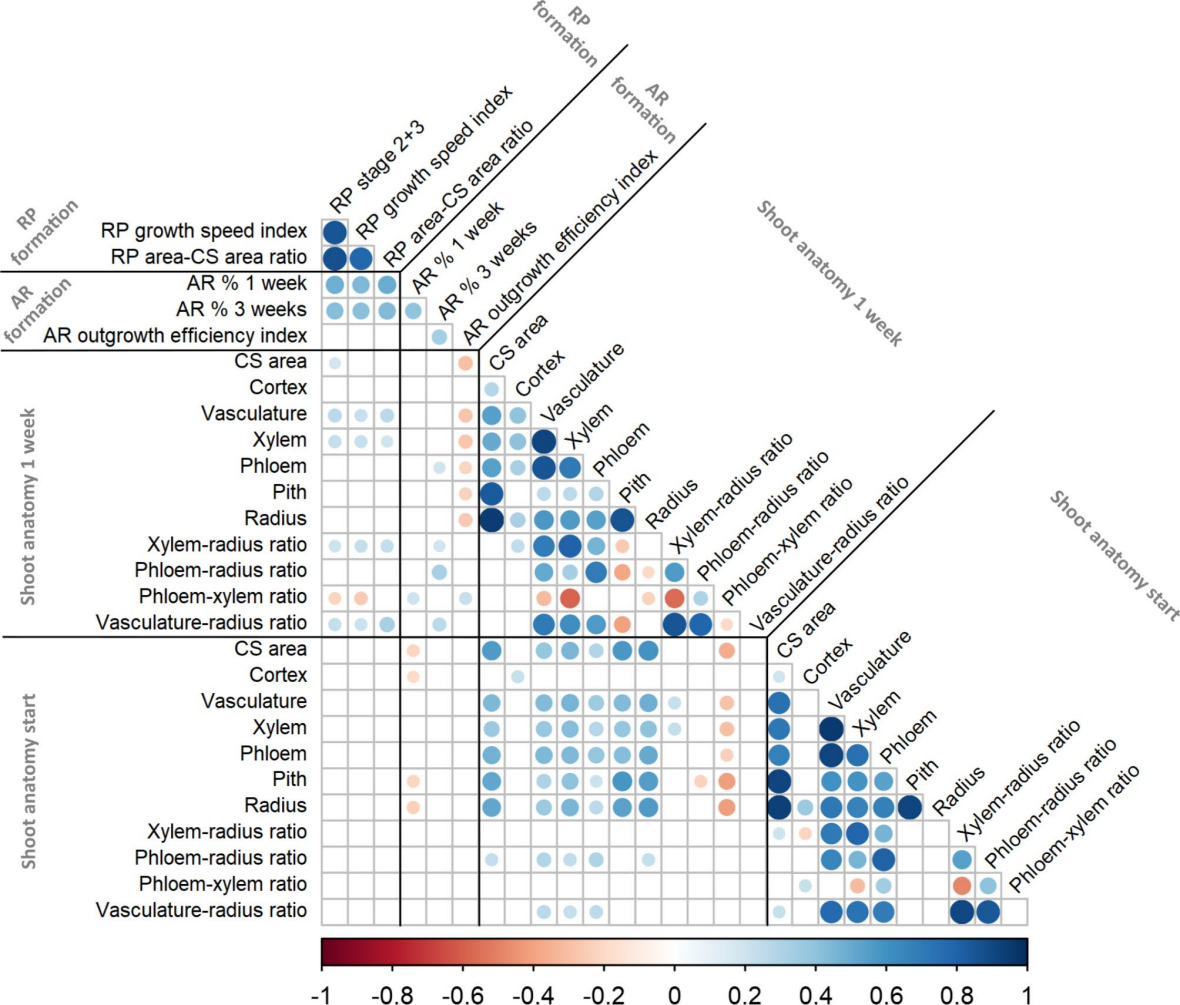
Pearson´s correlation coefficient matrix for root primordium (RP) and adventitious root (AR) formation and anatomical shoot characteristics. Pearson´s correlation coefficients between RP formation traits after 1 week, AR formation traits after 1 and 3 weeks, and anatomical shoot base characteristics at the start of the rooting experiments and after 1 week of cultivation on rooting medium for 106 tested genotypes are indicated. Coloured circles are only shown in the case of significant correlations (*p* < 0.05). The correlation coefficients and *p* values are shown in Supplementary Table S4 and Supplementary Table S5, respectively. Abbreviations: AR, adventitious root; CS, cross-section; RP, root primordium.

To link anatomical data, early RP data and AR formation, correlations between these datasets were analysed using Pearson´s correlation coefficients (Fig. 4). All RP characteristics were significantly and positively correlated with rooting percentage at 1 and 3 weeks (Fig. 4, Supplementary Tables S3 and S4). In addition, correlations between RP formation and shoot anatomy traits at 1 week, in particular vascular traits, tended to be positive, but negative correlations were observed for the phloem-xylem ratio with RP stages 2 + 3 and the RP growth speed index (Fig. 4). The correlations between the AR traits and shoot anatomical characteristics with the vascular system were mostly positive, with the exception of the AR outgrowth efficiency index, i.e., the ratio of rooting percentage after 3 weeks divided by the RP formation percentage after 1 week, which showed a predominantly negative relationship (Fig. 4). The correlations between different anatomical shoot characteristics at the beginning and after 1 week were mostly positive (Fig. 4). A more detailed correlation matrix including additional AR and RP formation traits is presented in Supplementary Fig. S4.

### Marker‒trait associations

**Fig. 5:**
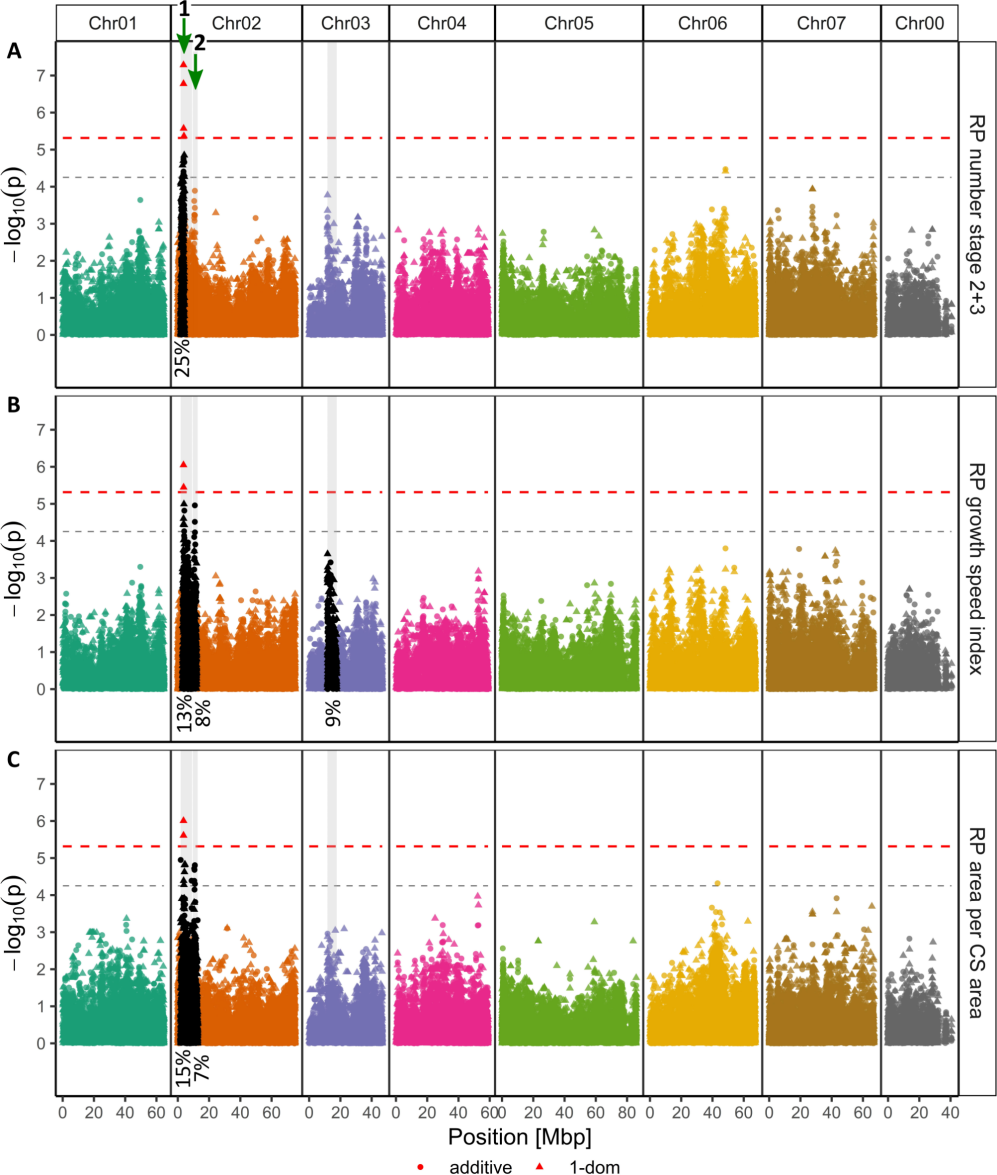
Manhattan plots for the associations of root primordium (RP) formation traits after 1 week of cultivation on hormone-free rooting medium. The results of marker‒trait associations and (**A**) RP number stage 2 + 3, (**B**) RP growth speed index and (**C**) RP area per cross-section (CS) area were analysed with an additive model (●) or a simplex dominance model (▲) and are shown as –log_10_ of the SNP´s specific *p* value. The x-axis shows the positions with respect to the seven *Rosa chinensis* chromosomes^23^ (Chr01-Chr07) in megabase pairs (Mbp). Chr00 covers contigs with SNPs that have not yet been mapped. The horizontal dashed red line indicates the M.eff-corrected *p* value significance threshold of 5.31 for the additive model, and the dashed black line indicates 80% of this threshold (4.25). SNPs reaching the model-specific significance thresholds are shown in red. The calculated widths of distinct peaks are highlighted with grey backgrounds. The black points indicate SNPs lying within a peak region and forming a distinct peak for the trait. The percentage values below the peaks represent the peak contribution (*R*^*2*^) to the variation in a certain trait.

**Fig. 6:**
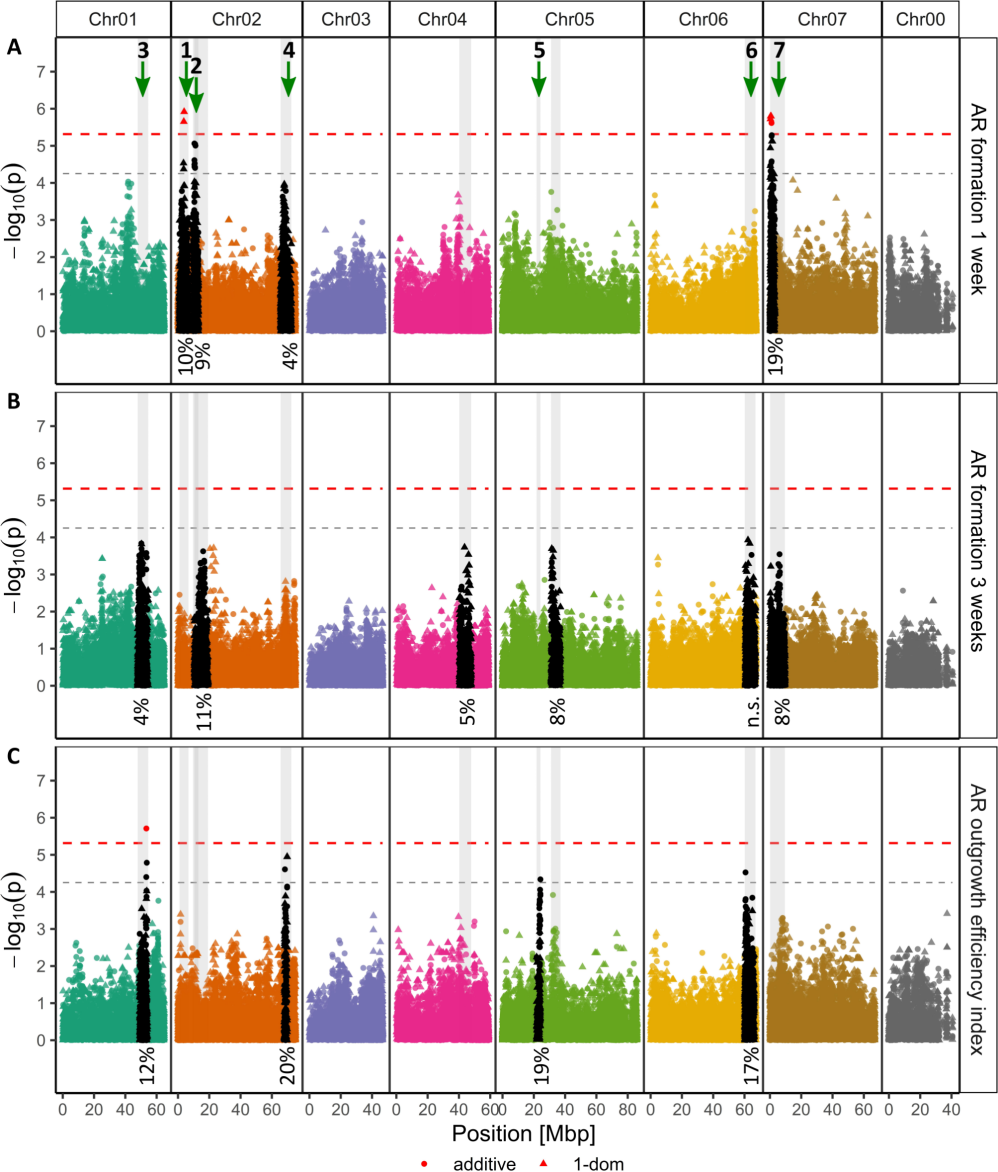
Manhattan plots for the associations of adventitious root (AR) formation after 1 and 3 weeks and the AR outgrowth efficiency index. The results of marker‒trait associations and (**A**) AR formation (%) after 1 week, (**B**) AR formation (%) after 3 weeks and (**C**) the AR outgrowth efficiency index were analysed with an additive model (●) or a simplex dominance model (▲) and are shown as –log_10_ of the SNP´s specific *p* value. The x-axis shows the positions with respect to the seven *Rosa chinensis* chromosomes^23^ (Chr01-Chr07) in megabase pairs (Mbp). Chr00 covers contigs with SNPs that have not yet been mapped. The horizontal dashed red line indicates the M.eff-corrected *p* value significance threshold of 5.31 for the additive model, and the dashed black line indicates 80% of this threshold (4.25). SNPs reaching the model-specific significance thresholds are shown in red. The calculated widths of distinct peaks are highlighted with grey backgrounds. The black points indicate SNPs lying within a peak region. The percentage values below the peaks represent the peak contribution (*R*^*2*^) to the variation in a certain trait.

Association mapping (AM) was performed for selected RP formation traits, focusing on RP number in stages 2 + 3, the RP growth speed index and the ratio between the RP area and cross-sectional area (Fig. 5). The AM results for AR formation after 1 and 3 weeks and for the AR outgrowth efficiency index are shown in Fig. 6, whereas those for each RP stage separately and all stages in sum are shown in Supplementary Fig. S5 and the AR formation after 2 weeks and the RP number per shoot in Supplementary Fig. S6. For each of the three RP traits, significantly associated SNPs were identified, all of which were located within a distinct peak at the beginning of Chr02 (Fig. 5A-C). Regarding the number of stage 2 + 3 RP, four SNPs showed a significant association. Among them, the SNP RhMCRND_28270_148Q had the highest significance (-log_10_(*p*) = 7.29) and the largest calculated dominant effect of 1.4 stage 2 + 3 RP (Fig. 5A; Table 1). Sequence analysis revealed that the SNP was derived from a sequence for a *homedomain-like superfamily protein* (*R. chinensis*) or, more precisely *early flowering MYB protein* or *hrs1 homolog4* (*HHO4*; *A. thaliana*) (Table 1). This SNP also showed a significant association with the RP growth speed index (-log_10_(*p*) = 6.05; effect = 0.344) and RP area per CS area (-log_10_(*p*) = 6.01; effect = 0.07). These were the greatest calculated effects recorded for the two significant SNPs for each of these traits (Table 1). Similarly, the other SNPs significantly associated with RP number stage 2 + 3 were also significantly associated with other traits: RhK5_1048_1857Q, located in the sequence for a *leucine-rich repeat protein kinase family protein* (RP area per CS area); RhK5_98_1449P, located in *pleiotropic drug resistance 4* (*PDR4*; RP growth speed index; AR formation 1 week); and RhK5_977_1943P, located in *abnormal inflorescence meristem* (*AIM1*; AR formation 1 week) (Figs. 5 and 6).

**Table 1:** SNPs significantly associated with root primordium (RP) and adventitious root (AR) formation traits, effect sizes and corresponding genes and gene models. Effect sizes were calculated and are expressed in the specific unit of the respective trait. The gene loci and models for rose were extracted from the *R. chinensis* ‘Old Blush’ genome^23^, and those for *Arabidopsis* were obtained from the reannotated Araport11 reference genome^24^.

Trait	Model	SNP-Marker	Chromosome	Position [Mbp]	-log(*p*)	Effect	Peak Nr	Loci	Gene model
RP number stage 2 + 3	1-dom alt	RhK5_1048_1857Q	Chr02	3.652	6.78	1.172	1	RC2G0048000	Leucine-rich repeat protein kinase family protein
								AT2G26730.1	no full name available
	1-dom ref	RhMCRND_28270_148Q	Chr02	3.635	7.29	1.379	1	RC2G0047700	Homeodomain-like superfamily protein
								AT2G03500.1	EARLY FLOWERING MYB PROTEIN, HRS1 HOMOLOG4
	1-dom alt	RhK5_98_1449P	Chr02	3.713	5.57	0.986	1	RC2G0048800	pleiotropic drug resistance 4
								AT2G26910.1	PERMEABLE CUTICLE 1, ATP-binding cassette G32, PLEIOTROPIC DRUG RESISTANCE 4
	1-dom alt	RhK5_977_1943P	Chr02	3.917	5.37	0.869	1	RC2G0050700	Enoyl-CoA hydratase/isomerase family
								AT4G29010.1	ABNORMAL INFLORESCENCE MERISTEM
RP growth speed index	1-dom ref	RhMCRND_28270_148Q	Chr02	3.635	6.05	0.344	1	RC2G0047700	Homeodomain-like superfamily protein
								AT2G03500.1	EARLY FLOWERING MYB PROTEIN, HRS1 HOMOLOG4
	1-dom alt	RhK5_98_1449P	Chr02	3.713	5.45	0.258	1	RC2G0048800	pleiotropic drug resistance 4
								AT2G26910.1	PERMEABLE CUTICLE 1, ATP-binding cassette G32, PLEIOTROPIC DRUG RESISTANCE 4
RP area per CS area	1-dom ref	RhMCRND_28270_148Q	Chr02	3.635	6.01	0.070	1	RC2G0047700	Homeodomain-like superfamily protein
								AT2G03500.1	EARLY FLOWERING MYB PROTEIN, HRS1 HOMOLOG4
	1-dom alt	RhK5_1048_1857Q	Chr02	3.652	5.61	0.058	1	RC2G0048000	Leucine-rich repeat protein kinase family protein
								AT2G26730.1	no full name available
AR formation 1 week	1-dom ref	RhMCRND_3811_1472P	Chr07	0.312	5.72	16.5	7	RC7G0004000	Peptidoglycan-binding LysM domain-containing protein
								AT5G08200.1	no full name available
	1-dom alt	RhMCRND_3597_1734Q	Chr07	0.459	5.8	13.3	7	RC7G0006800	Homeodomain-like superfamily protein
								AT1G18330.2	EARLY-PHYTOCHROME-RESPONSIVE1, REVEILLE 7
	1-dom ref	RhK5_2844_1239Q	Chr07	0.694	5.8	13.0	7	RC7G0010000	purple acid phosphatase 17
								AT3G17790.1	purple acid phosphatase 17
	add	RhMCRND_2205_1106Q	Chr07	1.007	5.62	-9.7	7	RC7G0015200	HCP-like superfamily protein
								AT1G18260.1	EMS-mutagenized bri1 suppressor 5
	1-dom alt	RhK5_98_1449P	Chr02	3.713	5.65	4.6	1	RC2G0048800	pleiotropic drug resistance 4
								AT2G26910.1	PERMEABLE CUTICLE 1, ATP-binding cassette G32, PLEIOTROPIC DRUG RESISTANCE 4
	1-dom alt	RhK5_977_1943P	Chr02	3.917	5.92	4.4	1	RC2G0050700	Enoyl-CoA hydratase/isomerase family
								AT4G29010.1	ABNORMAL INFLORESCENCE MERISTEM
AR outgrowth index	add	RhK5_7671_730P	Chr01	53.430	5.71	-57.8	3	RC1G0440200	Protein of unknown function
								AT2G39580.2	no full name available

Furthermore, SNPs showing a significant association with AR formation at 1 week were located at the beginning of Chr07 (Fig. 6): RhMCRND_3811_1472P in the sequence of *peptidoglycan-binding LysM domain-containing protein*; RhMCRND_3597_1734Q in a *homeodomain-like superfamily protein* (*R. chinensis*) or, more precisely, *early-phytochrome-responsive 1* (*REV7*; *A. thaliana*); RhK5_2844_1239Q in the sequence of *purple acid phosphatase 17*; and RhMCRND_2205_1106Q in an *HCP-like superfamily protein* (*R. chinensis*), namely, *EMS-mutagenized bri1 suppressor 5* (*HRD3A*; *A. thaliana*) (Table 1). These four SNPs had absolute effect sizes ranging from 9.7% (RhMCRND_2205_1106Q) to 16.5% (RhK5_98_1449P, RhK5_977_1943P), thus showing effects two to four times greater than those of the two previously mentioned SNPs on Chr02. In addition, a significantly associated SNP was found for the AR outgrowth efficiency index on Chr01 at approximately 53 Mbp, namely, RhK5_7671_730P, but the best BLAST result of the corresponding sequence was annotated to the sequence of a gene for a protein of unknown function (Table 1). No significantly associated SNPs resulted from the AM for AR formation after 3 weeks.

### Allele dosage effects for selected significantly associated SNPs

**Fig. 7:**
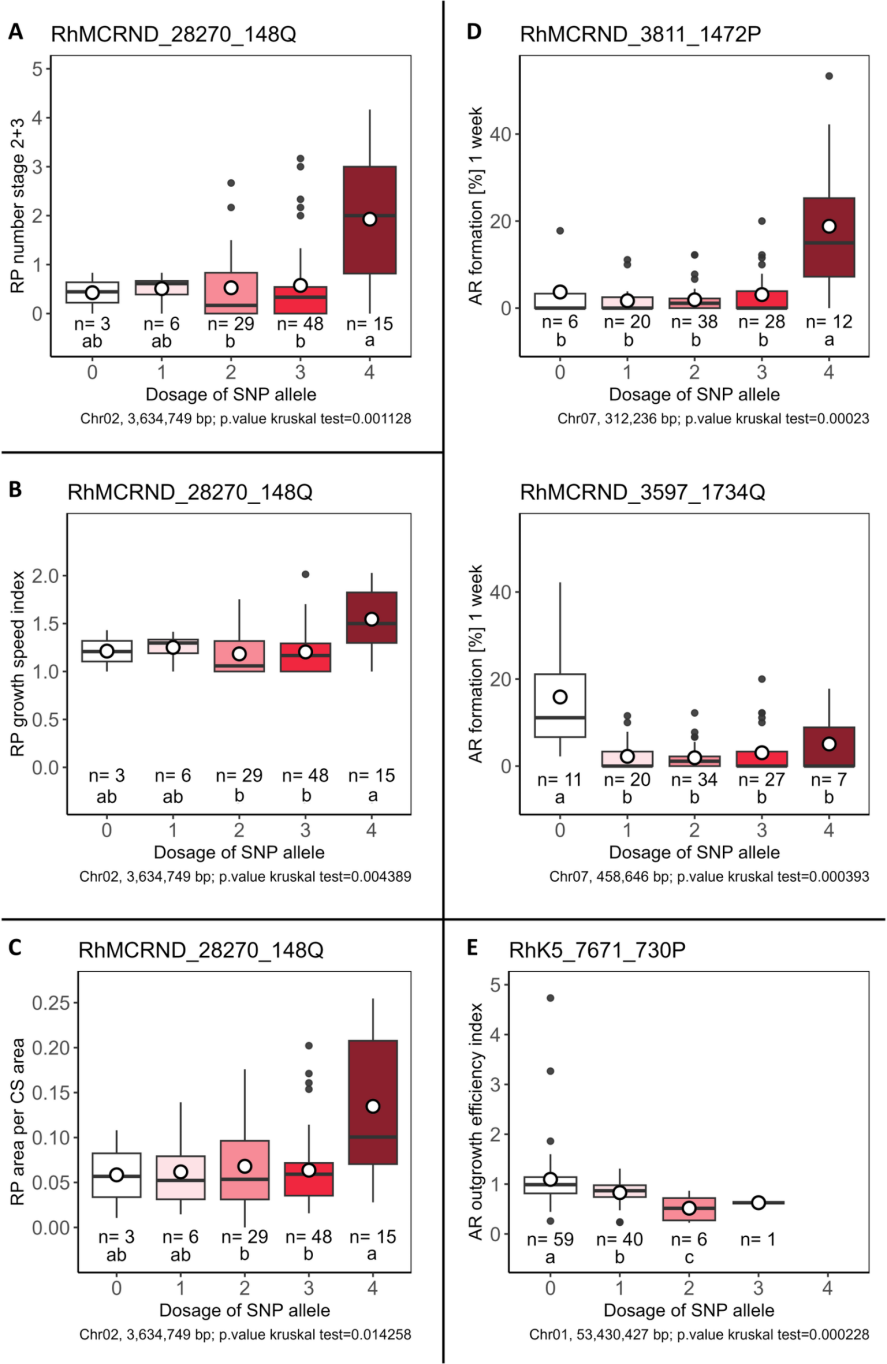
Effects of allele dosage configurations for selected significantly associated SNPs with different traits. The SNPs with the highest absolute effects calculated in *GWASpoly*^25^ of all significantly associated SNPs for each trait were selected. Allele dosage effects are shown for RP number stage 2 + 3 (**A**), RP growth speed index (**B**), RP area per CS area (**C**), AR formation after 1 week (two SNPs with the highest effects out of the comparatively large number of significantly associated SNPs) (**D**), and the AR outgrowth efficiency index (**E**). Letters indicate significant differences between allele dosage groups calculated via Fisher´s LSD criterion for *p* < 0.05 under consideration of the Holm‒Bonferroni adjustment; n indicates the number of individuals for each allele dosage group. AR = adventitious root, CS = cross-section, RP = root primordium.

Allelic discriminant plots for selected significantly associated SNPs with the highest effects for the traits from Table 1 show notable allelic dosage discriminations (Fig. 7). For the SNP RhMCRND_28270_148Q, which is associated with all RP formation traits (Table 1), quadruplex genotypes consistently showed the highest values in all three cases (Fig. 7A-C) and significantly higher values than the triplex and duplex allele dosage groups (ADGs). For AR formation (%) at 1 week, the SNPs RhMCRND_3811_1472P (effect 16.5%) and RhMCRND_3597_1734Q (effect 13.3%) showed relatively high effects (Table 1). Analysis of allele dosage effects revealed greater mean rooting percentages at 1 week for quadruplex (18.8%) genotypes than for all other ADGs for the RhMCRND_3811_1472P SNP (Fig. 7D). Conversely, for the SNP RhMCRND_3597_1734Q, significantly greater values were observed for nulliplex genotypes (15.9%) than for all other ADGs (Fig. 7D). For the AR outgrowth efficiency index, the SNP RhK5_7671_730P was the only SNP significantly associated with this trait (Table 1). The allele dosage plot showed significant differences in pairwise comparisons between the ADGs, but only three ADGs were considered due to the low number of triplex or quadruplex genotypes (Fig. 7E).

### Localisation of candidate genes involved in RP and AR formation in the identified peaks

With the aim of identifying additional genomic regions significantly contributing to the genotypic variation in the complex traits of RP and AR formation, we focused on distinct peaks reaching at least 80% of the significance threshold. This resulted in one (RP number stage 2 + 3) to three (RP growth speed index) peak regions for RP traits and four (AR formation 1 week; AR outgrowth efficiency index) to six (AR formation 3 weeks) peak regions for AR traits (Figs. 5 and 6). The global peak width ranged from 2.4 Mbp (AR outgrowth efficiency index; Chr02; 21.8 Mbp-24.2 Mbp) to 9.4 Mbp (AR formation 1 week and 3 weeks; Chr07; 0.1Mbp-9.5 Mbp) (Supplementary Table S6). Figures 5 and 6 show the contributions of these peaks/quantitative trait loci (QTLs) to the variation in each trait, taking into account all the detected peaks associated with the trait. For RP traits, the contribution of a single peak ranged from 7% (RP area per CS area) to 25% (RP number stage 2 + 3). For AR formation, contributions of 4% (AR formation at 1 week and 3 weeks) to 20% (AR outgrowth efficiency index) were recorded. The respective contributions were greater when fewer peaks were detected for a trait. Overall, seven peaks of interest located on five different chromosomes were further explored (Figs. 5 and 6; Supplementary Table S6). Within these peaks, promising potential candidates were searched for in the top 10% of all SNPs, i.e., SNPs showing the most substantial effects, as calculated by *GWASpoly*^25^. To reduce the number of SNPs, we selected only SNPs with at least four ADGs represented by a minimum of three individuals and with a significant difference for the trait between the ADGs. Maximum value normalisation further reduced the number of candidate SNPs, considering only those SNPs with at least 50% of the maximum effect size for a trait, resulting in 86 SNPs being located in sequences of 69 different *Rosa* gene homologues (Supplementary Table S7). These included homologues for genes previously identified as associated with AR formation, such as the auxin-associated genes *PIN-formed 1* (*PIN1*), *KANADI 1* (*KAN1*) and *inositol-polyphosphate 5-phosphatase 13* (*5PTASE13*) (Supplementary Table S7).

Finally, we screened the seven peaks for genes previously mentioned in the reviews of Guan et al. (2015)^26^, Druege et al. (2019)^11^ or Li (2021)^27^ that are involved in AR formation at different developmental stages. We assigned 16 rose gene homologues for phytohormone-related genes, such as *YUCCA2* (*YUC2*), *auxin response factor 16 (ARF16*), or the auxin efflux carrier *PIN1*; 27 homologues for cell division-/cell proliferation-related genes, such as several *NAC domain-containing protein*s (*NAC*) or *cyclin A* (*CYCA*); and 7 homologues for carbohydrate-related genes, such as *hexokinase* (*HXK*) or *sucrose synthase 1* (*SUS1*), to the genomic region of the seven selected peaks (Supplementary Table S8).

## Discussion

For the first time, a large set of 106 rose genotypes was characterised for both the ability to form RP and ARs in vitro and the stem anatomy at the bases of the microshoots. The data generated revealed genotypic variation and allowed correlations to be calculated between shoot anatomy and RP/AR formation. The data were also used to perform GWAS for traits of interest, focusing on the early stages of AR formation, with the aim of identifying genomic regions that contribute to the high genotypic variation in these traits.

The observed variation in AR formation (Figs. 1, 2 and 3; Supplementary Figs. S2, 3) is consistent with observations in previous studies on AR formation in rose using either cuttings^8,10,28^ or in vitro shoots cultivated on IBA-containing rooting medium^10^. However, the traits for root fresh mass and AR formation after two weeks were excluded from the analyses, as the results for the reference genotype ˈVendelaˈ indicated that these traits exhibited high sensitivity for the experimental conditions (Supplementary Fig. S2). The novel histological data on early RP formation and anatomical shoot characteristics allowed several insights: RP always originate from vascular tissue, which was previously described for rose cuttings rooted in vivo^16^ (Fig. 2). Interestingly, a high number of RP does not necessarily imply high AR formation for one or the same genotype (Fig. 2). In addition, dividing the RP into three developmental stages allowed the speed of RP development to be described by the RP growth speed index. The newly introduced AR outgrowth efficiency index connects RP with AR formation. For superior RP formation, the value decreases. However, for superior AR formation, the value increases. Consequently, this index has the potential to identify genomic regions that could be involved in regulating the emergence of developed RP. Therefore, peaks 3, 4, 5 and 6 were selected for further analysis (Fig. 6). Genes of the *NAC* family were most strongly represented in these peaks, and interestingly, an *NAC* gene homologue has been described to be involved in cell elongation of *Rosa hybrida* petals but also other organs, such as roots^29^.

Numerous significant correlations were found among RP, AR and anatomical traits (Fig. 4, Supplementary Fig. S4). RP and AR formation after 1 and 3 weeks showed strong correlations with each other (Fig. 4). The results for correlations involving anatomical parameters were of particular interest. Although only single and very weak correlations with RP and AR formation could be detected for anatomical characteristics at the beginning of the rooting period, the situation changed for the correlations involving shoot anatomy after 1 week (Fig. 4). Here, correlations with the dimensions of the vascular tissues, especially the xylem, were detected (Fig. 4). Similarly, Dokane et al. (2014)^20^ reported a greater phloem-to-xylem ratio in rooted shoots than in nonrooted shoots in *Rhododendron*. In our study, we observed negative correlations of the phloem-xylem ratio with the number of RP at stages 2 + 3 and the RP growth speed index but positive correlations with AR formation after 1 week and the AR outgrowth efficiency index (Fig. 4). Interestingly, a SNP located in peak 7 on Chr07 in the coding region of *KAN1* had an allele dosage effect on AR formation after 1 week (Supplementary Table S7). KAN1 in vasculature tissue was shown to negatively regulate the polar distribution of the auxin efflux carrier protein PIN1^30^. This resulted in an altered auxin distribution with inhibition of cambial activity during vasculature development^30^. Thus, positive correlations between vasculature traits and RP/AR formation could be attributed to elevated auxin levels in regions where RP are formed.

For the first time, we performed a GWAS using histological data for RP/AR formation with the aim of investigating the early time points of AR formation. As RP of more developed stages appeared to have greater correlations with AR formation (Supplementary Fig. S4; Table S2) and AM identified more distinct genomic regions for them, we focused on analyses of RP stages 2 + 3, the RP growth speed index and the ratio of RP area to cross-sectional area (Fig. 5; Supplementary Fig. S5). In addition, particularly the results for the separate AM of stages 2 and 3 (Supplementary Fig. S5) did not differ severely and more distinct peaks were observed for the sum of the two RP stages. Furthermore, we observed more peaks, each with a lower contribution to the observed variation, for traits for later AR developmental stages than for RP formation (Figs. 5 and 6). AR formation is known to be a highly complex quantitative trait^17^, which is supported by the results of this study. Our AM results indicate that the contribution of the individual genomic regions to the overall variation decreased at later time points, indicating either that genetic processes act mainly at earlier time points or that more genetic factors with smaller genetic contributions each act at later time points. As our small panel of genotypes allows only the detection of large-effect QTLs, we cannot differentiate/distinguish between these two possible explanations. However, the complex process of AR formation could be successfully dissected to focus on single aspects by introducing indices. The AR outgrowth index led to more distinct peaks in the Manhattan plots than did simple AR formation percentages (Fig. 6C peaks 3 and 6 compared to Fig. 6B, peak 4 compared to Fig. 6A). Similarly, the efficiency of RP outgrowth (AR outgrowth efficiency index) resulted in more pronounced peaks, indicating genomic regions associated with this index compared to those associated with RP or AR formation alone.

As an outcome of our GWAS, nine SNPs located in three peak regions (peaks 1, 3 and 7; Figs. 5 and 6; Table 1) showed significant associations, partly for several traits. Four SNPs each were assigned to peaks 1 (Chr02) and 7 (Chr07), and one SNP was assigned to peak 3 (Chr01) (Table 1). The majority of these SNPs are located in genes with known functions that allow particular interesting links to their potential involvement in the regulation of AR formation. The SNP RhMCRND_28270_148Q is located in the coding sequence of the MYB protein HHO4. Interestingly, the MYB HHO2 has been reported to be involved in IBA-mediated lateral root formation^31^. Consequently, a possible role of HHO4 in AR formation is conceivable but needs to be proven through functional studies. RhK5_98_1449P is located in *PDR4*, which is essential for cuticle development^32^. Interestingly, as another member of the *PDR* family, PDR9 was found to be involved in auxin efflux, which is essential for auxin distribution, especially in the early stages of AR formation^14,33^. The SNP RhK5_977_1943P is localised in *AIM1*, which is known to be involved in root meristem activity via salicylic acid biosynthesis and reactive oxygen species levels in *Oryza sativa*^34^, and increased expression of this gene was noted during AR formation in *Cucumis sativus*^35^. The SNP RhMCRND_3597_1734Q is located at the peak on Chr07 and can be assigned to the coding sequence for *REV7*, which is involved in circadian regulation, and the closely related homologue *REV1* regulates diurnal free auxin levels^36^. Interestingly, the significantly associated SNPs identified for several traits were also reflected in positive correlations between these traits, particularly between all RP traits and AR formation after 1 week (Fig. 4; Table 1). In summary, in addition to the possible role of gene homologues in AR formation, these SNPs are valuable candidates for the development of allele-specific PCR markers for the selection of RP/AR formation ability in independent populations. Such a marker has already been developed for a SNP located in the coding region of *SAC9*, allowing selection for the AR formation ability of cuttings under greenhouse conditions^8^.

A more detailed analysis of peaks that reached at least 80% of the significance threshold should increase the scope for selecting further promising candidate SNPs. Interestingly, some of the seven selected peaks (peaks 1, 5, and 6; Supplementary Table S7) identified in this study overlapped with the positions of peaks identified in previous GWASs for AR formation traits in vivo^8,10^. Among the large number of additional SNPs with effect sizes for different traits (Supplementary Table S7), many are located in gene homologues directly associated with AR formation or at least similar processes such as lateral root formation. Examples include BIG and PIN1, which are both involved in auxin transport^37^, and KAN1, which regulates auxin biosynthesis and transport^38^. In addition, SNPs located in gene homologues of *FAB1B* and *5PTASE13* should be mentioned, both of which are involved in phosphoinositide (PI) signalling^39^. PIs are involved in various growth and regulatory processes^39^. In a previous study, we developed an allele-specific PCR marker for selection for AR formation in vivo that is located in the coding region of the PI phosphatase *SAC9*^8^. This finding supports the hypothesis that PIs, as signalling molecules, may also play an important regulatory role in the processes of AR formation.

Finally, we searched for homologues of genes that have already been described to be involved in different processes and phases of AR formation^11,26,27^ and colocalised them in the defined seven peak regions. We divided the identified genes into three groups: phytohormone-related genes, cell division-/proliferation-related genes and carbohydrate-related genes (Supplementary Table S8). The increased number of homologues for certain genes was noticeable. For example, homologues for *YUC2* (2), *CYCA* (4) and *HXK* (3) were found in both peaks 1 and 6. Most homologues were detected for *NAC* (14) in peaks 3, 4, 6 and 7. YUC2 is involved in auxin biosynthesis. CYCAs regulate the cell cycle and are upregulated in the early induction phase of AR formation in several species, and HXK is upregulated during the induction of the formation of a new carbohydrate sink^11^. The localisation of such gene homologues in peak regions analysed in our AMs emphasises the relevance and contribution of these genomic regions to AR formation in rose.

This study presents combined data on AR formation, RP formation and anatomical characteristics of the shoot base for more than 100 rose genotypes for the first time. Our results indicate that indices for the differentiation of the vasculature can be predictors of the development of RP/ARs. Calculating indices integrating different time points of AR development not only improved the GWAS sensitivity but also allowed the correlation of anatomical data to the AR formation capacity. By association mapping, we found several SNPs associated with individual measures and indices correlated with AR formation. Among those SNPs, some are linked to or derived from candidate genes, such as *KAN1*, which is involved in the differentiation of the vascular system. Other genomic regions harboured several *NAC* homologues that have been reported to be involved in cell elongation as well as regions with genes involved in phosphoinositide signalling.

Our study therefore provides a starting point for further genetic and functional genomic analyses based on the identified candidate gene regions.

## Methods

### Plant material and in vitro culture conditions

A set of 81 cut rose and 25 garden rose genotypes collected from two existing association panels (previously described by Schulz et al. (2016)^40^ and Wamhoff et al. (2023)^8^) was used for GWAS on traits related to RP development and AR formation in vitro. Garden rose plants already have been established in vitro for previous experiments^41^. For the cut rose genotypes, nodal segments were collected from greenhouse-grown plants located at Rosen Tantau (25436, Uetersen, Germany) that were surface disinfected (30 s in 70% EtOH, 2 min in NaClO, 3 times 5 min in autoclaved deionised H_2_O). The explants were cultivated in medium consisting of full-strength MS salts and vitamins^42^ supplemented with 231 µM FeEDDHA to replace FeEDTA, 2.21 µM BAP (6-benzylaminopurine), 0.29 µM GA_3_ (gibberellic acid), 30 g L^−1^ sucrose, and 8 g L^−1^ Plant Agar (Duchefa, Harlem, Netherlands) for in vitro establishment and subsequent shoot proliferation. For genotypes displaying intense outgrowth of endophytic bacteria, 2 mL L^−1^ plant preservative mixture (PPM, Plant Cell Technology, Washington, USA) was added to shoot proliferation medium (Supplementary Table S1). For root induction, apical shoots 1 to 1.5 cm in length were cultivated on hormone-free rooting medium consisting of half-strength MS macro- and microelements and full-strength MS vitamins^42^, 20 g L^−1^ sucrose, and 7.5 g L^−1^ plant agar (Duchefa, Harlem, Netherlands). The media were autoclaved at 121 °C and 2,000 hPa for 20 min. The shoots were cultured for five and for rooting experiments for three weeks under fluorescent light tubes with a photoperiod of 16 h. The photon flux density (PAR) was 40 µmol m^−2^ s^−1^, and the temperature was 24 °C (± 2 °C). Shoot proliferation experiments were conducted in 250-mL polypropylene vessels (Plastikbecher.de GmbH, Giengen, Germany), and rooting experiments were performed in 150-mL glass jars with twist-off lids and filter papers.

### AR formation experiments

AR formation was monitored in vitro in glass jars for a rooting period of three weeks. For each genotype, five jars with six shoots each were analysed in three to five independent rooting experiments (Supplementary Table S1). Due to the large total number of explants, the rooting experiments were divided into 42 independent experiments with two to 18 genotypes per experiment (Supplementary Table S1). In 27 experiments, the ˈVendelaˈ genotype was included as a reference genotype, with 99 genotypes occurring at least once in the same experiments (Supplementary Table S1).

After 1 and 2 weeks of cultivation on rooting medium, AR formation (yes/no) was evaluated nondestructively. In addition to AR formation, the number of ARs per shoot was determined in a destructive manner at the end of the rooting experiments after 3 weeks.

### Histological analysis of shoot bases

For histological analysis, six shoots each were rooted in two repetitions in glass jars on rooting media as previously described and in parallel with the rooting experiments. After 1 week, the shoots were removed from the rooting medium, and the leaves were removed. The shoot bases were fixed in alcohol-formalin‒acetic acid fixation solution (7 parts of 96% EtOH, 2 parts of deionised water, ½ of 37% formaldehyde, and ½ of 100% acetic acid) and stored at 4 °C until further processing. As controls, two representative shoots were fixed directly at the start of rooting experiments for histological analyses.

### Paraffin embedding and cross-sectioning procedure

The fixed shoot bases were rinsed in deionised water and incubated in 70% EtOH at RT overnight. The samples were dehydrated in solutions with increasing EtOH concentrations (70%, 80%, 90%, and 96% EtOH (v/v) for 10 min, 15 min, 30 min, and 30 min, respectively) and incubated in 100% isopropanol for 30 min each. Afterwards, dehydrated samples were infiltrated with the xylene substitute ROTI^®^Clear (Carl Roth GmbH + Co. KG, Karlsruhe, Germany) twice for 30 min each, followed by infiltration in a 1:1 mixture of xylene substitute and paraffin (ROTI^®^Plast, Carl Roth GmbH + Co. KG, Karlsruhe, Germany) (1:1, v/v) at 65 °C. Then, the samples were infiltrated two times in pure ROTI^®^Plast for 40 min each at 65 °C. All infiltrations were performed under a vacuum of 11 kPa. Paraffin-infiltrated shoot bases were embedded in polyvinyl chloride embedding moulds (7 × 7 × 5 mm, PLANO GmbH, Wetzlar, Germany) in ROTI^®^Plast. A mesh of polypropylene with a mesh size of 1–2 mm (depending on the explant size; Franz Eckert GmbH, Waldkirch, Germany) was used to stabilise the stem base in the mould in an upright position during the cooling process on ice. Cross-sections were cut 5 μm to 10 μm thick with a rotation microtome (Hydrax M 55, Zeiss, Oberkochen, Germany). Cross-sections fixed on glass slides were deparaffinised in xylene substitute (twice, 10 min each) and rehydrated in a decreasing EtOH series (96%, 80%, 70%, 60% EtOH, 10 min each) followed by two steps in deionised water for 5 min each. Rehydrated cross-sections were serially RAA-stained with Rhodamine B solution (1%, dissolved in 50% EtOH, Kremer Pigmente GmbH & Co. KG, Aichstetten, Germany) for 10 min, aqueous Acriflavine solution (1% premixed, MOPRHISTO GmbH, Offenbach a.M., Germany) for 10 s, and then aqueous Astrablue (1%, premixed, MOPRHISTO GmbH, Offenbach a.M., Germany) for 2 min (the staining procedure was modified after Wacker (2006)). The cross-sections were rinsed in deionised water two to three times following every staining step. Finally, the sections were incubated in 100% isopropanol for 1 min. RAA-stained cross-sections were analysed microscopically using a VHX-7000 digital microscope (Keyence Deutschland GmbH, Neu-Isenburg, Germany). For each genotype, cross-sections of three shoot bases from two repetitions each were prepared for data collection after 1 week of rooting. To determine shoot anatomy characteristics at the start of rooting experiments, cross-sections of two independent shoot bases were analysed directly before the start of the rooting experiments.

### Image measurements and data processing

In total, 671 shoot bases/images harvested after 1 week (five to twelve images per genotype, 20 images for the reference genotype ˈVendelaˈ; see Supplementary Table S2) and 226 shoot bases/images harvested before transfer to rooting medium (two to three images per genotype, nine images for the reference genotype ˈVendelaˈ; see Supplementary Table S3) were analysed using VHX-7000_97F Communication software (version 1.4.23.17; KEYENCE Deutschland GmbH, Neu-Isenburg, Germany). The anatomical characteristics of all harvested shoot bases, such as the cross-sectional area of the whole shoot base and the radial expansion of the pith, xylem, phloem, and shoot cortex, were measured.

Additionally, for every shoot base harvested after 1 week of rooting, the area of every present RP/AR was measured, and the number of RP was counted, differentiating three stages of RP development: dome-shaped RP (stage 1), RP with its own vascular system (stage 2), and fully developed AR emerging from the shoot cortex (stage 3). Furthermore, the measurements were carried out twice for each shoot cross-section on orthogonally aligned lines originating from the centre of the cross-section. Radial vascular tissue expansion was calculated as the sum of xylem and phloem radial expansions. The shoot radius was calculated as the sum of radial expansions of the pith, xylem, phloem and shoot cortex. Based on the two measurements, the mean values per cross-section were calculated for pith, xylem, phloem, vascular tissue, and shoot cortex radial expansion and for the cross-section radius.

To measure and collect data from cross-sections with missing areas, different methods were used, as shown and explained in detail in Supplementary Fig. S7.

The RP data and anatomical characteristics per cross-section were used to calculate different indices for further characterisation and analyses: number of RP per cross-sectional area, ratio of RP area to cross-sectional area, xylem-to-radius ratio, phloem-to-radius ratio, vascular tissue-to-radius ratio, and phloem-to-xylem ratio. Furthermore, the RP growth speed index was calculated as follows:

$$RP\,growth\,speed\,index=\frac{{1*RP}_{stage1}+2*{RP}_{stage2}+3*{RP}_{stage3}}{{n}_{RPstage1+2+3}}$$

Additionally, an AR outgrowth efficiency index was calculated by dividing the number of AR-forming explants after 3 weeks of rooting (%) by the percentage of explants that formed RP after 1 week:

$$AR\,outgrowth\,\it{efficiency}\,index=\frac{{AR\%}_{3weeks}}{RP\%}$$

### Marker‒trait association analysis

SNPs were analysed with the 68 K WagRhSNP Axiom array^22^ for genotypes of garden and cut rose populations, and the raw data were further processed using the *R* packages* SNPpolisher*^43^ and *fitTetra*^44^ as previously described^8,40^. GWAS analyses were performed using the *R* package *GWASpoly*^25^ as previously described by Wamhoff et al. (2023)^8^. For discriminant analysis of principal components (DAPC), the *R* package *adegenet*^45^ was used. Minor allele frequency (0.05) and missing marker thresholds (0.1) resulted in 28,359 SNPs being included in GWAS analyses. Furthermore, *p* < 0.05 was used for the *M.eff* method (*R* package *GWASpoly*^25^), , leading to adjusted significance thresholds of 4.8*10^−6^ (log_10_(*p*) = 5.31) for additive, 6.5*10^−6^ (-log_10_(*p*) = 5.18) for 1-dom-ref, and 6.6*10^−6^ (-log_10_(*p*) = 5.19) for 1-dom-alt allele dosage trait relationship models. The results are displayed in Manhattan plots generated using the *R* package *ggplot2*^46^.

The statistical significance between allele dosage groups for significantly associated SNPs was assessed using the Kruskal‒Wallis test (*p* < 0.05). For pairwise differences between allele dosage groups, the post hoc Fisher´s least significant difference criterion (LSD) was used (*p* < 0.05, Holm‒Bonferroni adjustment). The effect sizes of SNPs following an additive and/or dominant model of association were computed as described previously in Wamhoff et al. (2023)^8^.

### Candidate SNP marker and gene homologue identification

For each peak indicating a genomic region of interest, the full width of the half-height maximum peak region was defined by the following four steps: (1) Extraction of the -log_10_(*p*)_maximum_ showing the most significant SNP in the peak region. (2) Calculation of the half-height maximum threshold by -log_10_(*p*)_maximum_/2. (3) Define the left and right border positions of the peak regions by SNPs with values between -log_10_(*p*)_maximum_ and -log_10_(*p*)_maximum_/2. (4) Calculation of the peak contribution to trait variance was performed using the function *fit.* All the QTLs (peaks) for a trait were subjected to backwards elimination in the *R* package *GWASpoly*^25^ .

Genes and their sequences localised in peak regions were extracted from the *Rosa chinensis* ‘Old Blush’ genome^23^ and blasted against the reannotated Araport11 *Arabidopsis thaliana* reference genome^24^ via the *R* package *rBLAST*^47^ to identify gene homologues. Conversely, to identify AR formation-associated genes within the peaks, sequences of genes that have been reviewed by Guan et al. (2015)^26^, Druege et al. (2019)^11^ or Li (2021)^27^ and are involved in various processes of AR formation were extracted from the Araport11 *Arabidopsis thaliana* reference genome and blasted against the *Rosa chinensis* ‘Old Blush’ genome.

Furthermore, SNPs with an absolute effect size from *GWASpoly* among the largest 10% for a trait and peak region were selected. At least four ADGs with a minimum of three individuals each were tested for allele dosage effects for a distinct trait using the Kruskal‒Wallis test and post hoc least significant difference (LSD) test, as previously described for significantly associated SNPs. For SNPs showing significant differences according to the Kruskal‒Wallis test and significant group differences according to the LSD test, the effect sizes in units of the distinctive traits were calculated. Furthermore, gene homologues for selected SNPs were determined.

### Data analyses

Pearson´s correlation coefficients for all measured AR formation traits were calculated using the *R* package *stats*^48^ and visualised in a correlation matrix plot using the *R* package *corrplot*^49^.

For the reference genotype ˈVendelaˈ, within the AR formation experiments, statistical analyses were performed to test for significant differences between individual experiments. Binomial rooting data were analysed using *generalised linear models* (GLMs) under the assumption of a binomial or quasibinomial distribution. Root numbers per rooted shoot were analysed using GLM under the assumption of a quasipoisson distribution. For both, the experiment was set as a fixed effect. When the deviance analyses showed a significant effect for the factor experiment, pairwise comparisons (Tukey, *p* < 0.05) were performed by using the *R* package *emmeans* (v1.8.4-1^50^) . Descriptive statistical visualisation was performed with the *R* package *ggplot2*^46^.

## Electronic supplementary material

Below is the link to the electronic supplementary material.

Supplementary Material 1

Supplementary Material 2

## Data Availability

All data are available at the Research Data Repository of Leibniz University Hannover under the following DOI: Wamhoff et al. (2024): Genotypic and phenotypic raw data for GWAS study Wamhoff et al. 2024. 10.25835/xvdntbqg.
