# Supplementary material for: Genome-wide association study (GWAS) analyses of early anatomical changes in rose adventitious root formation
Source: Sci Rep. 2024 Oct 23;14:25072. doi: 10.1038/s41598-024-75502-1 (PMC11499985; doi:10.1038/s41598-024-75502-1)
Supplement: Supplementary file 2 — Supplementary Material 2 [file 41598_2024_75502_MOESM2_ESM.pdf]

Supporting information

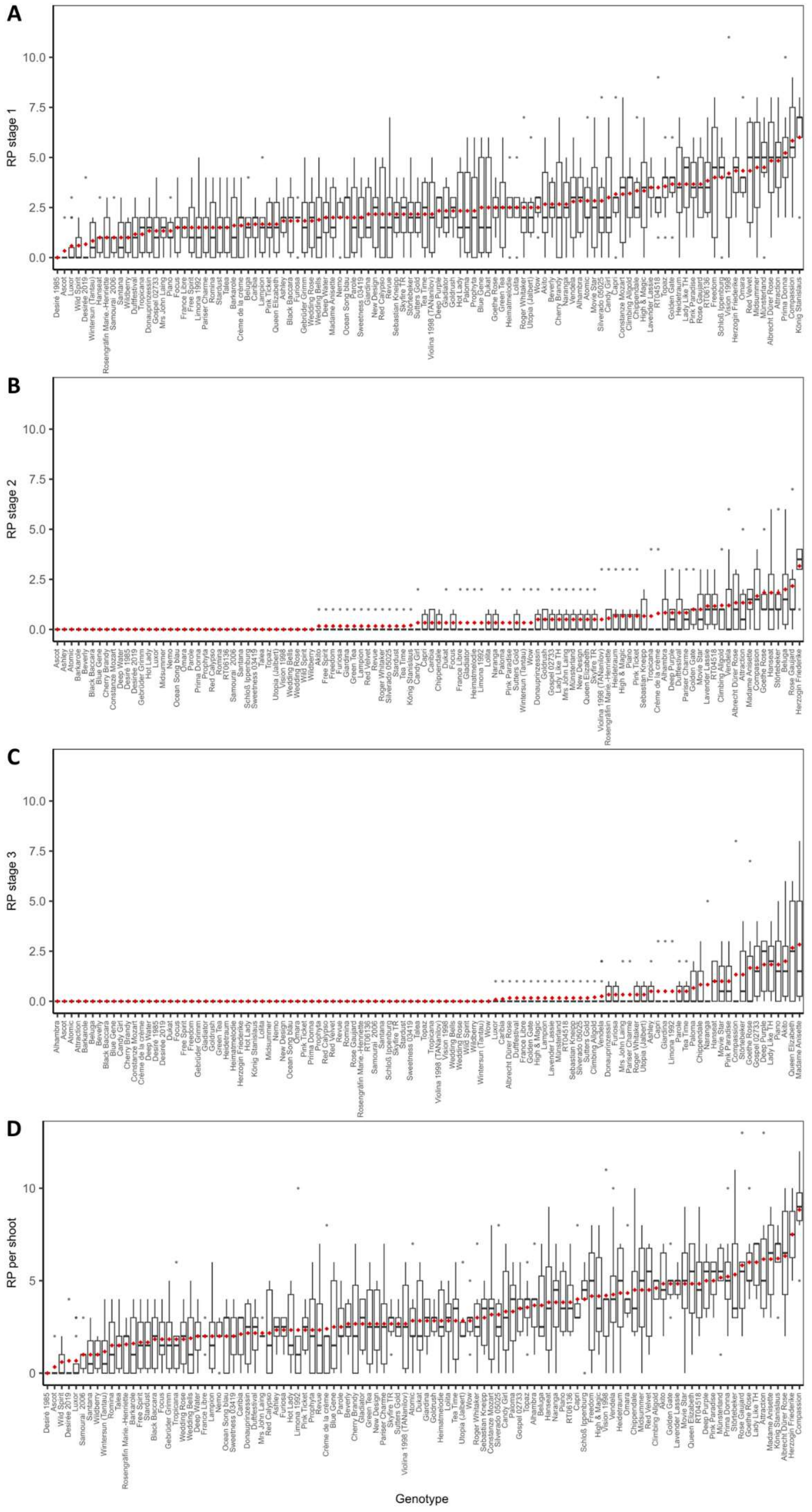

A
RP stage 1
10.0
7.5
5.0
2.5
0.0
B
RP stage 2
10.0
7.5
5.0
2.5
0.0
C
RP stage 3
10.0
7.5
5.0
2.5
0.0
D
RP per shoot
10
5
0
Genotype

Figure S1. **Root primordium (RP) formation of 106 tested rose genotypes after 1 week of incubation on hormone-free rooting medium.** Number of RP at (**A**) stage 1, (**B**) stage 2, (**C**) stage 3 and (**D**) the sum of all stages per analysed shoot base after 1 week of incubation on hormone-free rooting medium. Genotypes are ordered based on their means (◆). The replicate numbers per genotype are given in Supplementary Table S2.

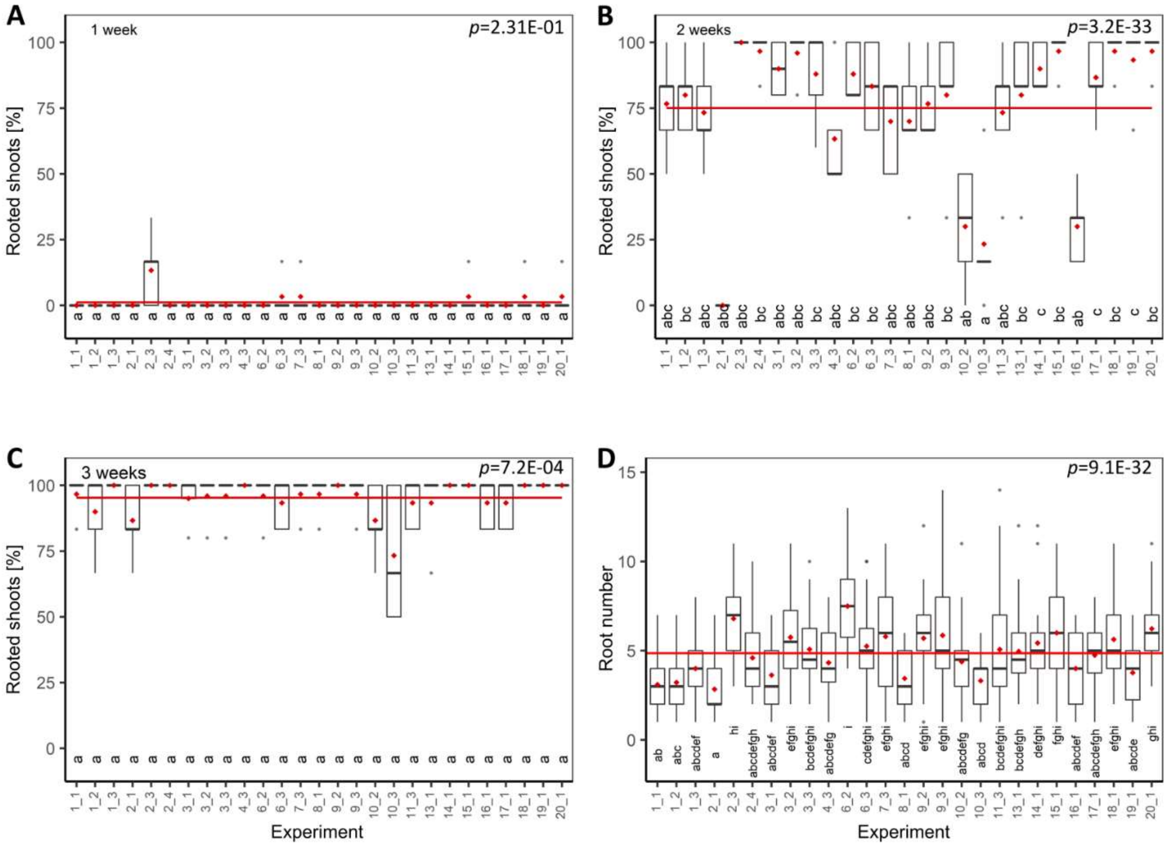

Figure S2. **Adventitious root (AR) formation characteristics for reference genotype 'Vendela'.** Rooting percentage after (**A**) 1 week, (**B**) 2 weeks, (**C**) 3 weeks and (**D**) root number per rooted shoot separated per experiment (ordered chronologically). ◆ shows the mean per experiment, the red line indicates the mean performance over all experiments. The *p* value shows the result of deviance analyses testing for an effect of the factor experiment, letters indicate significance groups as determined by *Tukey`s* post-hoc test at *p*<0.05.

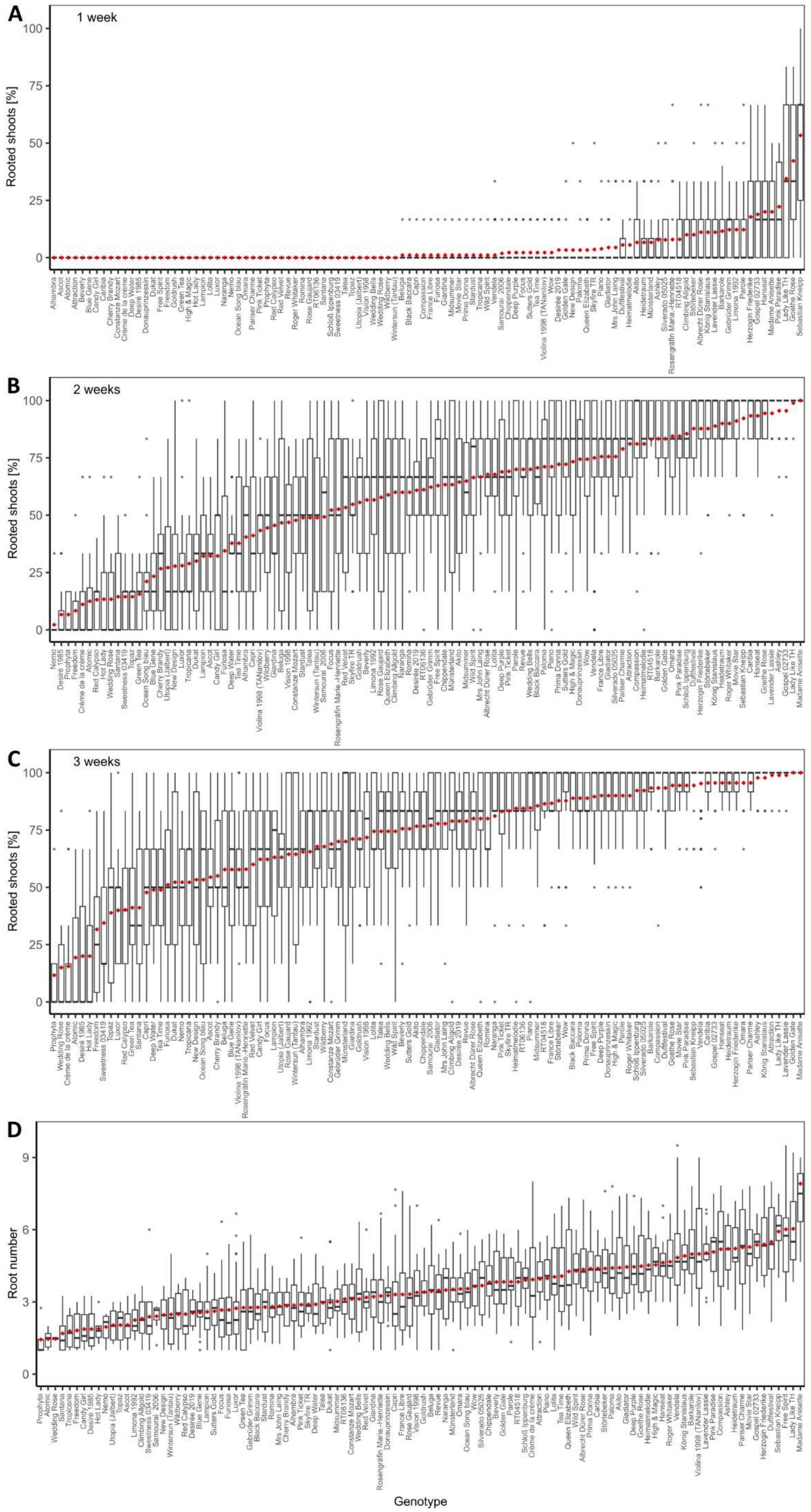

A
1 week
B
2 weeks
C
3 weeks
D
Rooted shoots [%]
Root number
0
25
50
75
100
3
6
9
Genotype

Figure S3. **Adventitious root (AR) formation characteristics of 106 tested rose genotypes incubated on hormone-free rooting medium.** Rooting percentage after (**A**) 1 week, (**B**) 2 weeks and (**C**) 3 weeks and (**D**) root number per rooted. Genotypes are ordered based on their means (◆). The replicate numbers per genotype are given in Supplementary Table S1.

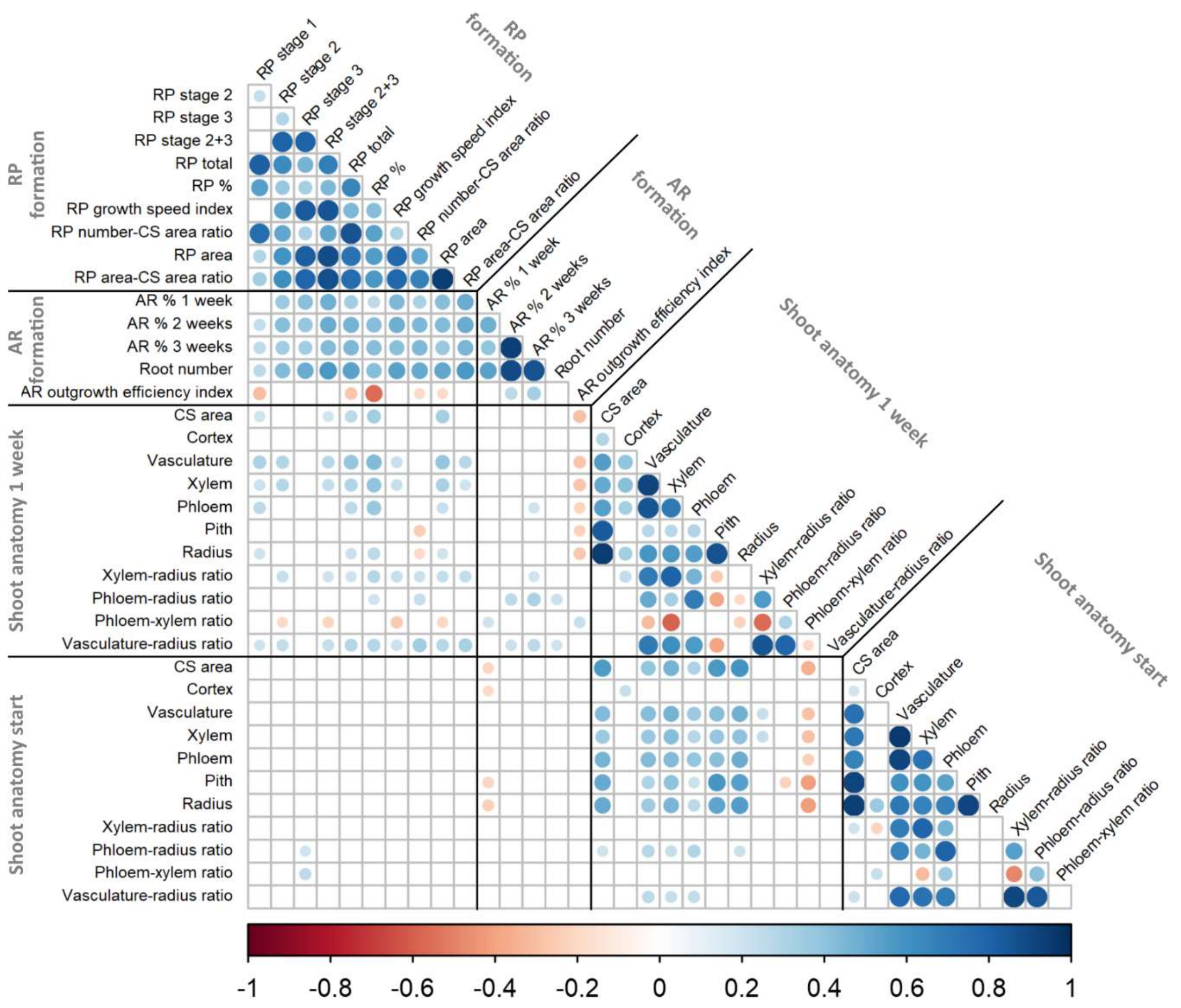

Figure S4**. Pearson´s correlation coefficient matrix for adventitious root (AR) and root primordium (RP) formation and anatomical shoot characteristics.** Pearson´s correlation coefficients between RP formation traits after 1 week, AR formation traits after 1, 2 and 3 weeks, and anatomical shoot base characteristics at the start of the rooting experiments and after 1 week of cultivation on rooting medium for 106 tested genotypes. Coloured circles are only shown in case of significant correlations ($p<0.05$). The correlation coefficients and *p* values are shown in Supplementary Table S4 and Supplementary Table S5, respectively. Abbreviations: AR, adventitious root; CS, cross-section; RP, root primordium.

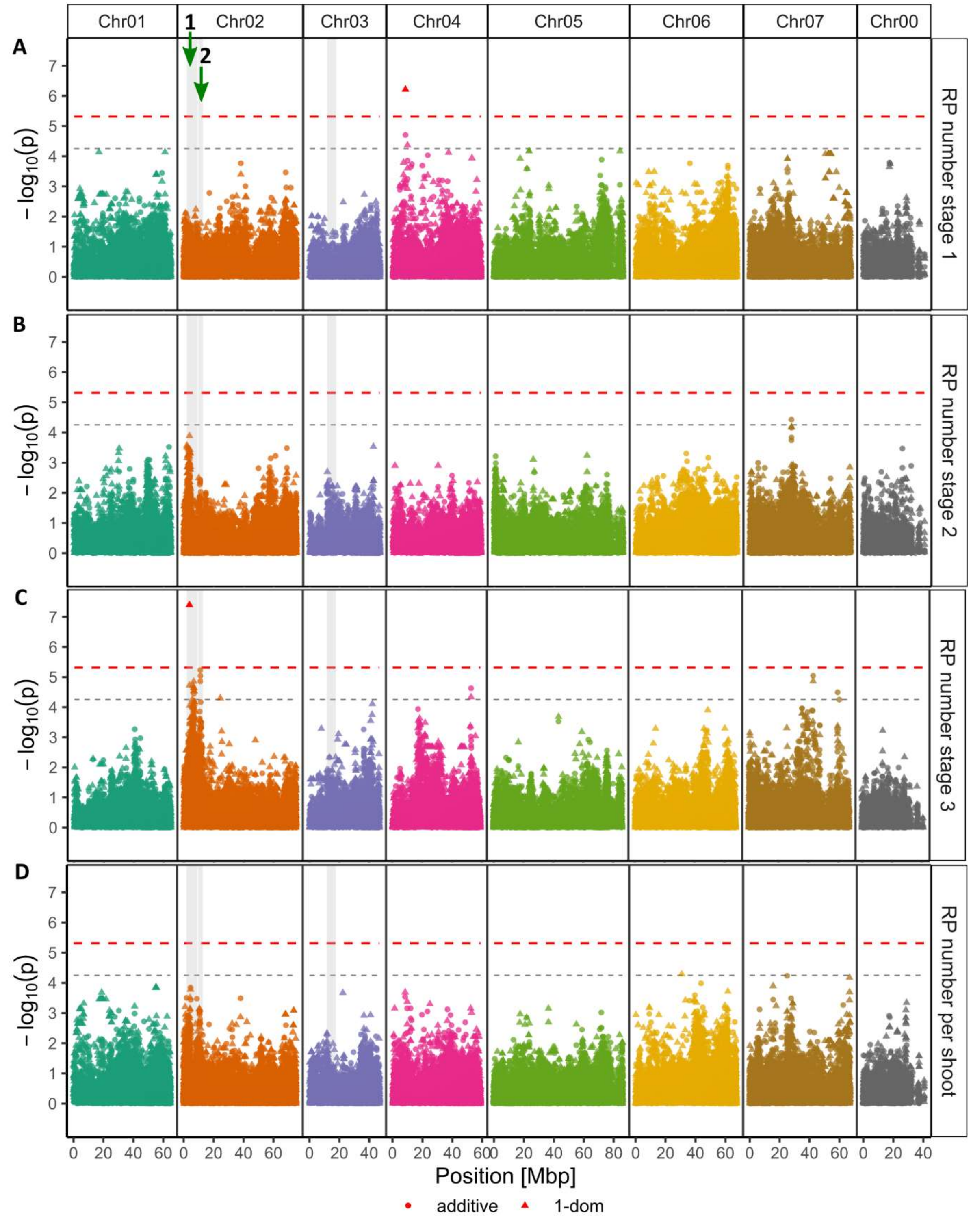

***Figure S5*. Manhattan plots for the associations of root primordium (RP) formation traits after 1 week of cultivation on hormone-free medium.** The results of marker-trait associations and (**A**) RP number stage 1, (**B**) RP number stage 2, (**C**) RP number stage 3 and (**D**) total number of RP per shoot base were analysed with an additive model (●) or a simplex dominance model (▲) and shown as $-\log_{10}$ of the SNP´s specific *p* value. The x-axis shows the positions with respect to the seven *Rosa chinensis* chromosomes[23] (Chr01-Chr07) in megabase pairs (Mbp). Chr00 covers contigs with SNPs that have not yet been mapped. The horizontal dashed red line indicates the M.eff corrected *p* value significance threshold of 5.31 for the additive model, and the dashed black line indicates 80% of this threshold (4.25). SNPs reaching the model-specific significance thresholds are shown in red. The calculated widths of distinct peaks are highlighted with grey backgrounds.

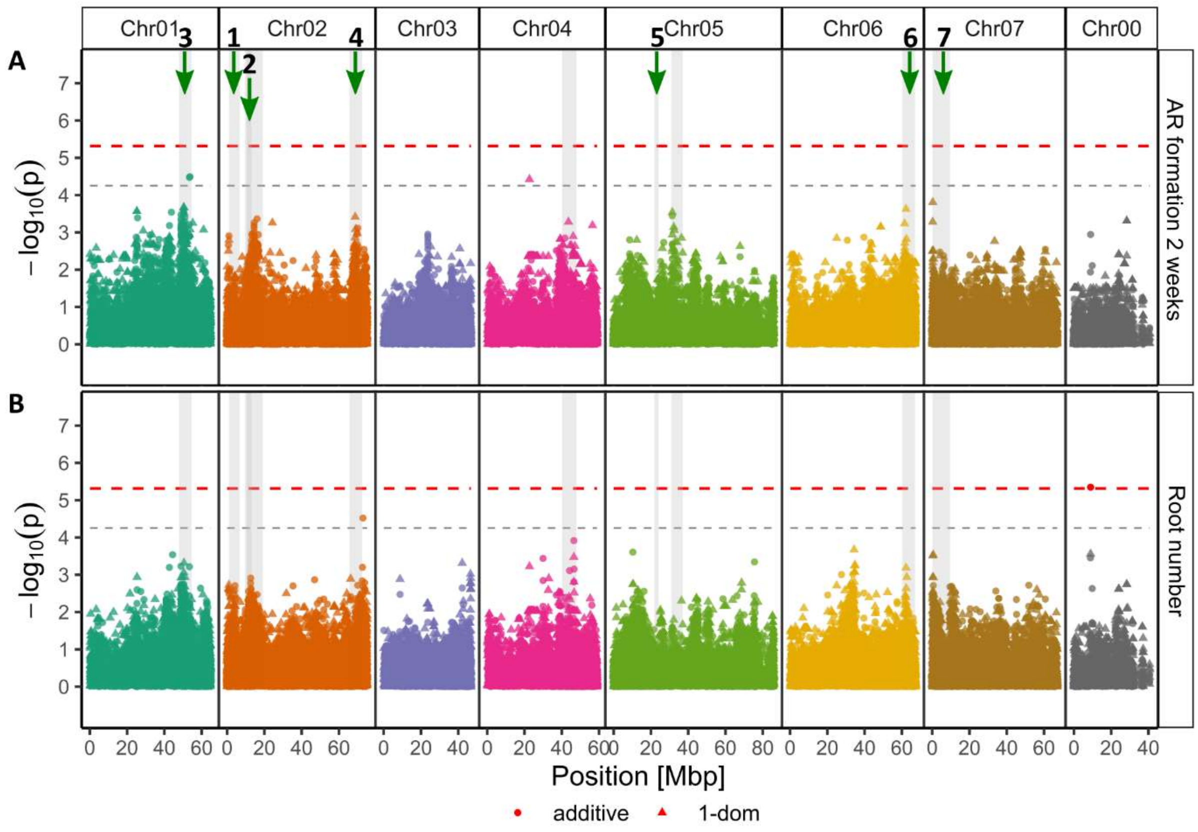

***Figure S6*. Manhattan plots for the association of adventitious root (AR) formation after 2 weeks of cultivation and for the root number per rooted shoot.** The results of marker-trait associations and AR formation traits (**A**) AR formation (%) after 2 weeks and (**B**) the root number per rooted shoot were analysed with an additive model (●) or a simplex dominance model (▲) and shown as $-\log_{10}$ of the SNP´s specific *p* value. The x-axis shows the positions with respect to the seven *Rosa chinensis* chromosomes[23] (Chr01-Chr07) in megabase pairs (Mbp). Chr00 covers contigs with SNPs that have not yet been mapped. The horizontal dashed red line indicates the M.eff corrected *p* value significance threshold of 5.31 for the additive model, and the dashed black line indicates 80% of this threshold (4.25). SNPs reaching the model-specific significance thresholds are shown in red. The calculated widths of distinct peaks are highlighted with grey backgrounds.

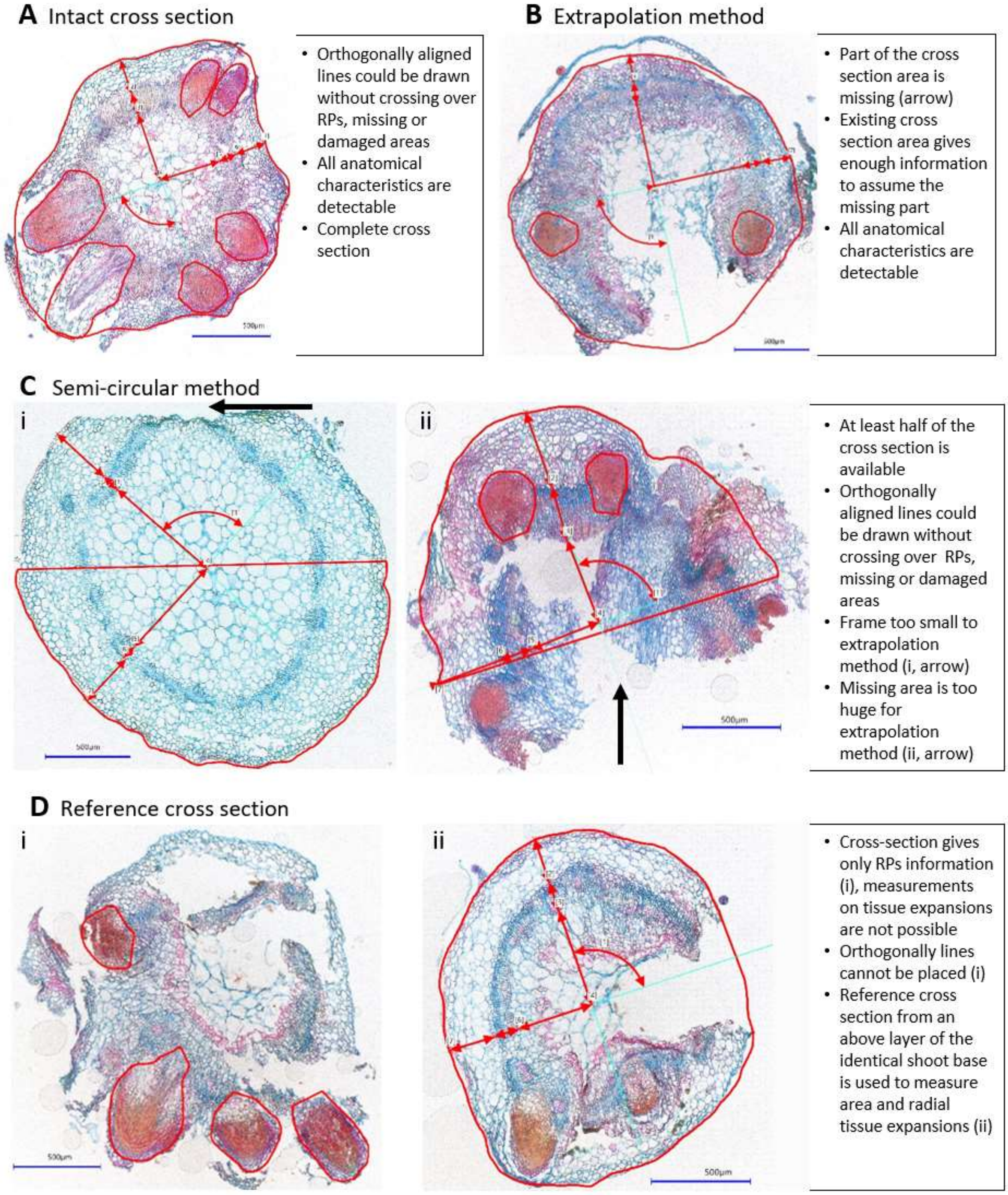

***Figure S7*. Graphical representation of the measurements and data collection on shoot cross-sections of different qualities.** Examples for data collection on (**A**) intact cross-sections, (**B**) cross-sections with missing areas by extrapolation method, (**C**) for half cross-sections by semi-circular method and (**D**) cross sections without possibility to measure anatomical characteristics. When the semi-circular method (**C**) was applied, only RP located within the measured semicircle were counted and doubled, to account for a representative RP number per determined complete cross-section. (**D**) was only applied in case no other method (**A-C**) could be applied to determine anatomical characteristics, i.e. radial expansions and cross-section area, and reference cross-section was taken from the same explant but from a higher level in the apical direction of the shoot.

Table S1. **Cut and garden rose genotypes tested within adventitious root (AR) formation experiments and in histological analysis for marker–trait association studies.** List of 81 cut rose and 25 garden rose genotypes tested in experiments and further cultivar-specific information. Column PPM gives information whether the plant material was subcultured on shoot proliferation medium supplemented with plant preservative mixture (PPM, Plant Cell Technology, Washington, USA). X indicates a genotype was tested within a distinct experiment. Cells were filled green as they were tested together with reference genotype 'Vendela' within the experiment. Abbreviations: CR, cut rose; GR, garden rose; N, no; Y, yes.

***Table S2*. Anatomical shoot base dimensions and additional root primordium (RP) formation data after 1 week of rooting.** Data is presented as mean ± standard deviation per genotype. The number of analysed shoots/images per genotype is given by “n shoots”, the number of shoots that showed RP are given as “n shoots RP forming”. Abbreviations: CR, cut rose; GR, garden rose.

Table S3. **Anatomical shoot base dimensions data at the start of rooting experiments.** Data is presented as mean ± standard deviation per genotype. The number of analysed shoots/images per genotype is given by “n shoots”. Abbreviations: CR, cut rose; GR, garden rose.

Table S4. **Pearson´s correlation coefficients for correlation analysis of adventitious root (AR), root primordium (RP) and shoot anatomy traits for the 106 tested genotypes.** Values are related to correlation matrices shown in Fig. 4 and Supplementary Figure S4. Cells are filled blue for traits that were analysed in GWAS. Abbreviations: AR, adventitious root; CS, cross-section; RP, root primordium.

Table S5. P **values for Pearson´s correlation analysis of adventitious root (AR), root primordium (RP) and shoot anatomy traits for the 106 tested genotypes.** Values are related to correlation matrices shown in Fig. 4 and Supplementary Fig. S4. Cells are coloured in green in case the correlation between the two related traits was significant ($p<0.05$). Abbreviations: AR, adventitious root; CS, cross-section; RP, root primordium.

Table S6. **Position and variance contribution information on peaks identified in marker-trait association analyses.** Peak positions for manually defined peak regions, calculated peak widths for single traits ($xmin_{peak}$, $xmax_{peak}$), for traits of same phenotypic data group (root primordium, RP; adventitious root, AR; $xmin_{group}$, $xmax_{group}$), and combined for all traits that have been analysed ($xmin_{total}$, $xmax_{total}$). Results from function fit.QTL analysing the contribution of QTLs to the variance of a trait by applying the function to every single QTL identified for a trait of interest. Positions are given in megabasepairs (Mbp).

Table S7. **Gene homologues located in peak regions and that contain SNPs with high allele dosage effects.** Loci for *R. chinensis* and SNPs´ position are based on the annotation of the *R. chinensis* genome[23], loci *A.t.* is based on the re-annotated Araport11 reference genome[24].

Table S8. **Predicted genes positioned in peaks 1-7 and previously shown to be involved in root primordium (RP) and adventitious root (AR) formation related processes.** Genes previously reviewed by Guan et al. (2015)[25], Druege et al. (2019)[11] or Li (2021)[26]. Genes are based on their function assigned to four different groups: group of auxin-, cell division- or cell proliferation-, ethylene- or carbohydrate-associated genes.
